# A Tale of Two Reductases: Extending the Bacteriochlorophyll Biosynthetic Pathway in *E. coli*

**DOI:** 10.1371/journal.pone.0089734

**Published:** 2014-02-21

**Authors:** Ilya B. Tikh, Maureen B. Quin, Claudia Schmidt-Dannert

**Affiliations:** Department of Biochemistry, Molecular Biology and Biophysics, University of Minnesota, St. Paul, Minnesota, United States of America; Institute of Enzymology of the Hungarian Academy of Science, Hungary

## Abstract

The creation of a synthetic microbe that can harvest energy from sunlight to drive its metabolic processes is an attractive approach to the economically viable biosynthetic production of target compounds. Our aim is to design and engineer a genetically tractable non-photosynthetic microbe to produce light-harvesting molecules. Previously we created a modular, multienzyme system for the heterologous production of intermediates of the bacteriochlorophyll (BChl) pathway in *E. coli*. In this report we extend this pathway to include a substrate promiscuous 8-vinyl reductase that can accept multiple intermediates of BChl biosynthesis. We present an informative comparative analysis of homologues of 8-vinyl reductase from the model photosynthetic organisms *Rhodobacter sphaeroides* and *Chlorobaculum tepidum*. The first purification of the enzymes leads to their detailed biochemical and biophysical characterization. The data obtained reveal that the two 8-vinyl reductases are substrate promiscuous, capable of reducing the C8-vinyl group of Mg protoporphyrin IX, Mg protoporphyrin IX methylester, and divinyl protochlorophyllide. However, activity is dependent upon the presence of chelated Mg^2+^ in the porphyrin ring, with no activity against non-Mg^2+^ chelated intermediates observed. Additionally, CD analyses reveal that the two 8-vinyl reductases appear to bind the same substrate in a different fashion. Furthermore, we discover that the different rates of reaction of the two 8-vinyl reductases both *in vitro*, and *in vivo* as part of our engineered system, results in the suitability of only one of the homologues for our BChl pathway in *E. coli*. Our results offer the first insights into the different functionalities of homologous 8-vinyl reductases. This study also takes us one step closer to the creation of a nonphotosynthetic microbe that is capable of harvesting energy from sunlight for the biosynthesis of molecules of choice.

## Introduction

Sunlight is an abundant and sustainable energy source that is captured by photosynthetic organisms and converted into chemical energy for growth and survival. Utilization of the photosynthetic machineries of light harvesting organisms plays an important role in the bioproduction of fuels and chemicals [1]–[4]. Engineering light capture and conversion into genetically tractable, nonphotosynthetic and robust microorganisms already used for industrial processes represents an alternative approach [5], [6]. Such designer microbes could be engineered to synthesize a range of valuable and novel compounds from inexpensive carbon sources where light-energy drives otherwise expensive synthetic reactions [7].

**Figure 1:**
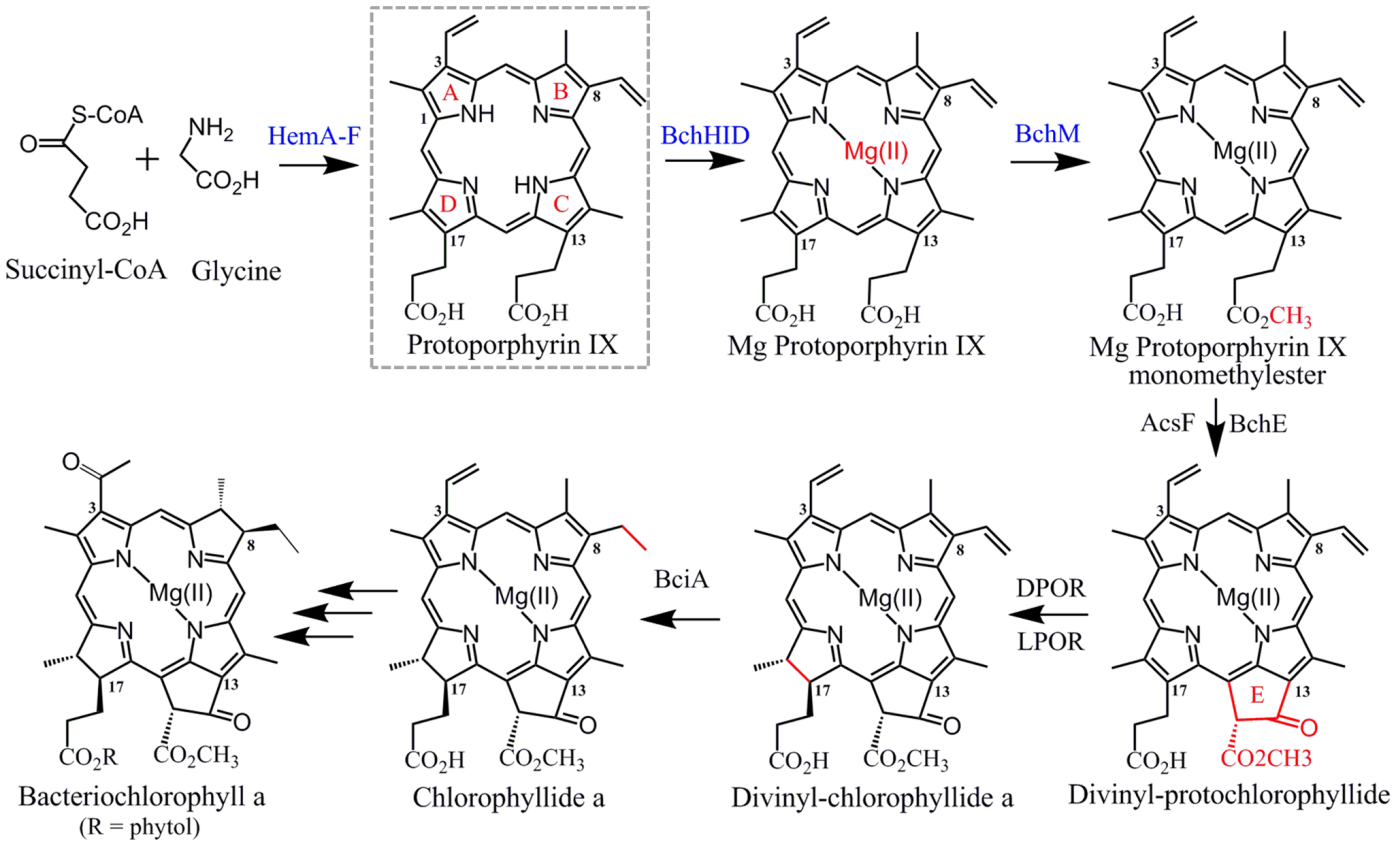
Engineered pathway design for the heterologous production of BChl in the non-photosynthetic host *E. coli*. Using succinyl-CoA and glycine as precursor molecules, expression of the heme pathway enzymes HemA-F in *E. coli* results in production of P^IX^ as the common intermediate of the heme and BChl biosynthetic pathways. Addition of the BChl enzymes magnesium chelatase (BchHID) and methyltransferase (BchM) yields MgP^IX^ and MgP^IX^ME in *E. coli* [11], [15]. Subsequent steps have not yet been functionally assembled in a heterologous system and depending on the enzymes substrate specificities, the order in which the enzymes operate may differ from the depicted pathway. Briefly, formation of the characteristic fifth E ring of chlorophylls is catalyzed by two unrelated and yet to be biochemically characterized cyclases AcsF (aerobic) [20] or BchE (anaerobic) [19]. The D pyrrole ring is reduced either by a light-dependent, nitrogenase-like (LPOR, three-subunit enzyme BchLNB) or a light-independent (DPOR) protochlorophyllide reductase; both enzymes have been biochemically characterized [23]–[26]. Reduction of the C8-vinyl group of BChl intermediates is catalyzed by the NADPH-dependent reductase BciA [27], [30] investigated in this study. Seven additional enzymatic steps are required for production of Bchl *a* [14].

The first steps on the path towards engineering a nonphotosynthetic microorganism able to harvest light-energy are to install either simple light-driven proton pumps [8] or more powerful photosynthetic reaction centers [5], [6]. Both systems require functional assembly of a biosynthetic pathway for carotenoid-derived pigments, and reaction centers also require (bacterio)chlorophyll ((B)Chl) pigments for function. While engineering of carotenoid pathways into various hosts has been shown [9], [10], complete reconstruction of a BChl biosynthetic pathway remains to be demonstrated, a formidable task owing to the complexities of the reaction pathway and enzymes involved (Fig. 1).

As a first step towards this goal, we created a modular system for the high level production of porphyrins, including protoporphyrin IX (P^IX^), by assembling genes involved in heme biosynthesis (HemA-F) in *E. coli* [11]. P^IX^ is the common intermediate between the heme and BChl biosynthetic pathways [12]–[14] and is committed to Bchl biosynthesis upon insertion of a central Mg^2+^ catalyzed by a multi-subunit magnesium (Mg-) chelatase enzyme complex BchHID (homologues of H, namely S and T, are present in some bacteria like the green sulfur bacterium *Chlorobaculum tepidum*) [15]. The chelatase subunit BchH interacts with the SAM-dependent methyltransferase BchM, which methylates MgP^IX^ at the C13-carboxyl group, resulting in MgP^IX^ monomethyl ester (MgP^IX^ME) [16]–[18]. Co-expression of BchSID and BchM from *C. tepidum* in our P^IX^ overproducing *E. coli* strain resulted in high level production of P^IX^, P^IX^ME, MgP^IX^ and MgP^IX^ME [15]. Detailed *in vitro* studies provided insights into enzyme interactions and kinetics and revealed that BchM also methylates P^IX^, resulting in the accumulation of the “dead-end” product P^IX^ME, which cannot be chelated by BchSID [15].

Following chelation and methylation of P^IX^, the characteristic fifth ring of the chlorin molecule is formed under anaerobic conditions by the radical-SAM cyclase BchE, or under aerobic conditions by AcsF, producing divinyl protochlorophyllide (DVP) [17], [19], [20]. Reduction of the D pyrrole ring of DVP to produce chlorophyllide is either catalyzed by a light-independent, nitrogenase like (DPOR) or by a light-dependent (LPOR) protochlorophyllide reductase [21]–[26]. An NADPH-dependent reduction of the C8-vinyl group to an ethyl group by 8-vinyl reductase BciA results in chlorophyllide *a* [27]–[30]. Beyond this, another seven polypeptides are required to complete the biosynthesis of bacteriochlorophyll *a* [14]. Fig. 1 shows the upper part of the BChl pathway; depending on the substrate specifities of the biosynthetic enzymes, the order in which they operate may differ from the sequence shown.

While some of these additional enzymes have been functionally expressed and biochemically characterized *in vitro* (e.g. LPOR and DPOR [23], [25], [31]), other steps of this complex pathway have only been elucidated by gene knockouts/deletions, complementation, and mutational studies [14], [32], [33]. Many of these enzymes form complexes, catalyze novel reactions and may interact with yet to be identified protein partners [34], making biochemical studies as well as heterologous pathway reconstitution particularly challenging. In our quest towards recombinant BChl biosynthesis, we report the extension of the BChl biosynthetic pathway in *E. coli* to include an 8-vinyl reductase. Recent studies have indicated that various homologues of the 8-vinyl reductase BciA are substrate promiscuous *in vivo* and can reduce the C8-vinyl group of different intermediates of the BChl pathway [35], [36]. We hypothesized that including an 8-vinyl reductase as the next step in our pathway would result in reduction of the C8-vinyl group of multiple BChl intermediates that do not have the fifth ring of divinyl protocholorophyllide (DVP) (Fig. 1), thereby possibly removing barriers to the efficient turnover of P^IX^ in our engineered system.

We demonstrate co-expression of the heme biosynthetic pathway in conjunction with *C. tepidum* BchSID and BchM with two separate homologues of BciA from *Rhodobacter sphaeroides* (*RS*BciA) [30] and *C. tepidum* (*CT*BciA) [27]. We discovered that while *CT*BciA is capable of reducing the C8-vinyl group of several different intermediates in the BChl pathway, *RS*BciA is surprisingly completely inactive in our recombinant system. We therefore conducted a full purification and *in vitro* characterization of the two BciA homologues to elucidate possible mechanisms for their different activities. Results show that both *RS*BciA and *CT*BciA are substrate promiscuous *in vitro*, however, the two enzymes exhibit very different catalytic turnover efficiencies. Biophysical characterization suggests that these differences may be related to different mechanisms of substrate binding. This study provides useful insights for BChl pathway design and another enzymatic step in the complex pathways leading to (B)Chls.

## Materials and Methods

### Materials

All chemicals were obtained from Sigma Aldrich (St. Louis, MO), unless otherwise stated. Restriction enzymes and DNA polymerases were purchased from New England Biolabs (Ipswich, MA) and were used according to manufacturers’ procedures. Protein ladder was purchased from Biorad (Hercules, CA).

### Bacterial Strains, Plasmids and Growth Conditions

All bacterial cultures were grown under aerobic conditions at 37°C with shaking at 220 rpm in Luria-Bertani (LB) medium supplemented with chloramphenicol (50 µg/ml), kanamycin (30 µg/ml), ampicillin (100 µg/ml), and streptomycin (50 µg/ml) as required for plasmid maintenance. The pET30a (+) vector was purchased from EMD Millipore (Billerica, MA). *R. sphaeroides* 2.4.1 and *C. tepidum* TLS were acquired from the ATCC collection (Manassas, VA). *E. coli* JM109 was used for all genetic manipulations and *E. coli* BL21 (DE3) was used for protein expression.

### Construction of Plasmids

Expression vectors containing *rsbciA* and *ctbciA* were constructed as follows: pET30-*rsbciA* was constructed by amplifying *bciA* (rsp_3070, GenBank Accession Number: 3721347) from *R. sphaeroides* 2.4.1 genomic DNA using the gene specific primers P1 (forward) and P2 (reverse) (Table S1), with the reverse primer introducing a His_6_ tag for purification purposes. Similarly, pET30-*ctbciA* was constructed by amplifying *bciA* (ct_1063, GenBank Accession Number: 1006951) from *C. tepidum* TLS genomic DNA using primers P3 and P4 (Table S1), again introducing a His_6_ tag for purification. The PCR products were digested with *Nde*I and *Not*I and were cloned into pET30a (+) which was digested with the same enzymes, and the sequence of the resulting plasmids was verified by DNA sequencing. Vector pCDFBB-*rsbciA* was constructed by amplifying *rsbciA* using primers P5 and P6, digesting with *Bgl*II and *Not*I and ligating into pCDFBB-GFP which was digested with the same enzymes. Plasmid pCDFBB-*bchM* was created by amplifying *bchM* from pCDF-*bchM* [15] using primers P7 and P8 and the resulting PCR product inserted into the *Bgl*II and *Not*I sites of pCDFBB. Similarly, *ctbciA* was cloned into the *Bgl*II and *Not*I sites of pCDFBB after amplification with P9 and P10, generating pCDFBB-*ctbciA*. Subsequent gene stacking of *rsbciA* and *ctbciA* with *bchM* was performed via standard BioBrick techniques, described elsewhere [37], generating plasmids pCDFBB-*bchM-rsbciA* and pCDFBB-*bchM-ctbchiA*. Similarly, *bchJ* (rsp_0280, GenBank Accession Number: 3719192) was amplified from *R. sphaeroides* 2.4.1 genomic DNA using primers P11 (forward) and P12 (reverse) containing *Bgl*II and *Not*I site, respectively. Following restriction enzyme digest, the PCR product was ligated into the same sites of pCDFBB to create pCDFBB-*bchJ*. *bchJ* was stacked with *bchM* and *rsbciA* in a pCDFBB backbone following standard BioBrick techniques, as described above. Plasmids used in this study are listed in Table S2.

### Sequence and Phylogenetic Analysis

All protein alignments were performed in MEGA5.1 [38] using the ClustalW algorithm [39]. The protein sequences were identified using NCBI BLAST [40] with *RS*BciA as the query and hit proteins limited those containing above 30% sequence identity. The phylogenetic tree was created in MEGA5.1 using the default parameters for the neighbor-joining algorithm [41] with a bootstrap test of phylogeny (500 replicates) [42]. Protein modelling was conducted using the automated mode in MODELLER v 9.12 [43] and models were visualized using PyMOL v 1.6 (Schrödinger, LLC).

### Biosynthesis of Protoporphyrin IX Derivatives in *E. coli*

The production of magnesium porphyrins in *E. coli* harboring plasmids pAC-*hemAD*, pBBRB-*hemEF*, pUCMOD-*SID* and pCDF-*bchM* has been described previously [11], [15]. Briefly, *E. coli* were transformed with the plasmids and were incubated at 30°C with shaking at 220 rpm in 4 ml of LB broth supplemented with chloramphenicol (50 µg/ml), kanamycin (30 µg/ml), ampicillin (100 µg/ml), streptomycin (50 µg/ml) and 1 mM MgCl_2_ for 48 hours. Porphyrins were extracted from the cells as follows: 0.25 ml of culture was centrifuged at 21000×*g* for 1 minute and the pellet was resuspended in 1 ml of a water:acetone:methanol mix at a 1∶7∶2 ratio. The cells were lysed and the pigments were extracted by vortexing for 20 seconds every 10 minutes over the course of two hours. Subsequently, the samples were centrifuged at 21000×*g* for 5 minutes to remove cell debris and the remaining supernatant was analyzed with an Agilent 1100 HPLC system equipped with an photodiode array detector as previously described [15]. Comparison of samples to the integrated peak areas of known concentrations of authentic P^IX^ and MgP^IX^ standards (Frontier Scientific, Logan, UT) was used to determine porphyrin concentrations. For determination of *in vivo* activity of BciA and BchJ, pigments were extracted from *E. coli* cultures as described above, with pCDFBB-*bchM* being replaced with a pCDFBB-*bchM* vector containing one of the BciA homologues, or BchJ.

### Protein Expression and Purification

*E. coli* cells were transformed with either pET30-*ctbciA* or pET30-*rsbciA*, and single colonies were used to inoculate 1 L LB broth supplemented with kanamycin (30 µg/ml). Protein expression was induced at an OD_600_ of 0.6 upon addition of isopropyl β-D-thiogalactopyranoside (IPTG) to a final concentration of 0.5 mM. The culture was incubated for an additional 16 hours at 30°C and cells were harvested by centrifugation. The cell pellet was resuspended in Buffer A (50 mM Tris-HCl, 100 mM NaCl, 5 mM imidazole, pH 8.0) and cells were lysed by sonication. The supernatant was clarified by centrifugation at 15 000×*g* for 20 minutes and the soluble portion was loaded onto a 5 ml HisTrap FF column (GE Healthcare, Piscataway, NJ) which was equilibrated with Buffer A. The protein was eluted from the column over a linear gradient to 100% Buffer B (50 mM Tris-HCl, 100 mM NaCl, 250 mM imidazole, pH 8.0) over 20 column volumes. The resulting fractions were analyzed by SDS-PAGE to assess protein purity. Fractions determined to be >95% pure were pooled and the protein concentration was determined by Coomassie Plus Bradford assay (Thermo Scientific, Rockford, IL).

### Size Exclusion Chromatography

Purified BciA was dialyzed overnight into Buffer C (50 mM Tris-HCl, 10% glycerol, 1 mM DTT, pH 8.0) using 3 kDa cutoff dialysis tubing. Size exclusion chromatography was performed at a flow rate of 0.5 mL/min by passing 30 µM BciA over a Superdex S200 10/300 GL column (GE Healthcare, Piscataway, NJ) equilibrated with Buffer C. Standard curves were prepared using standards of known molecular weights (Biorad, Hercules, CA).

### Circular Dichroism

Prior to structural analysis by CD, purified BciA was dialyzed into Buffer D (10 mM NaHPO_4_, 100 mM NaCl, pH 7.4). The secondary structure of BciA was analyzed using a J-185 spectrometer (JASCO, Easton, MD). Data were collected using 1 nm wavelength intervals from 200–260 nm using 7.5 µM of protein in a 1 mm pathlength cuvette. Conformational changes in metal porphyrins were monitored in 1 nm steps between the wavelengths of 350–500 nm upon addition of 20 µM metal porphyrin to BciA. Analysis of protein secondary structure content was performed with CDPro using the CONTIN method and SMP56 reference set [44].

### 8-vinyl Reductase Enzyme Assay

DVP (divinyl protochlorophyllide) was purchased from ChromaDex (Irving, CA). P^IX^ME and Mg-P^IX^ME were purified from *E. coli* cells as previously described [15]. Enzyme assays were carried out in Buffer E (50 mM Tris-HCl, pH 8.0). Final reaction volumes of 50 µL included 500 µM of either NADH or NADPH as a cofactor, substrate at the concentrations listed below, and 1 µM of purified BciA. In the case of control reactions, an equal volume of Buffer E was added in place of BciA. The reactions were incubated for 30 minutes at 37°C for DVP (2 µM) or 18 hours at 25°C for other porphyrin substrates (75 µM). Reactions were quenched by addition of 70 µL acetone. Precipitated protein was removed by centrifugation. The reaction products were analyzed on an Agilent 1100 HPLC system equipped with an photodiode array detector. Porphyrin derivatives were separated using a 25 mm Zorbax C-18 column (Agilent Technologies, Santa Clara, CA) using a gradient of 80%–100% methanol over 55 min at a flow rate of 1 ml/min, using an aqueous buffer (0.1 M ammonium acetate, pH 5.1) for reactions containing a mixture of porphyrins. DVP was resolved using isocratic conditions of 80% methanol and 20% aqueous buffer. For structural analysis, mass fragmentation spectra were monitored on a LCQ mass spectrophotometer equipped with an electron spray ionization (ESI) interface (Thermo Finnigan, USA) operating in the positive mode.

## Results

### Selecting BciA Homologues for Pathway Assembly

Our previously engineered, modular heme biosynthetic pathway in *E. coli* was assembled from *hemA-F* genes selected from diverse bacterial sources (*E. coli*, *B. subtilis*, *R. capsulatus* and *Synechocystis* sp. PCC 6803) based on available biochemical data and reported activities [11]. We then chose to extend the heme pathway with the first two steps of the BChl pathway comprised of BchHID(S,T) and BchM from the green sulfur bacterium *C. tepidum* [15]. Green bacteria like *C. tepidum* are unique in that they are able to produce different types of Chls and Bchls, and encode in their genomes several homologs (BchS, T) of the large subunit (BchH) of the magnesium chelatase, which may play a role in regulating the types of (B)Chls produced (see [15] for a comprehensive discussion). These are useful properties that may later become important for heterologous production of different (B)Chl structures.

To apply the same combinatorial rationale to the current BChl pathway extension, we searched all domains of life for suitable 8-vinyl reductase candidates. Using the recently described 8-vinyl reductase BciA from the well characterized model photosynthetic purple bacterium *R. sphaeroides* [30], [45] to guide our searches, we identified 38 putative BciA-like 8-vinyl reductases in photosynthetic organisms belonging to five domains (Fig. S1). Of those homologues identified, only five have been previously described in literature [27], [28], [30], [36], [46], and activity has been shown either by gene knockouts in the native host (*R. sphaeroides, C. sativus, A. thaliana, Z. mays*), or by heterologous expression and analysis of cell lysate constituents (*C. tepidum, A. thaliana*). A single report of the purification and characterization of an 8-vinyl reductase exists for *C. tepidum* [47]. Hence, insufficient information currently exists concerning 8-vinyl reductases to make an informed choice of the best enzyme(s) for our pathway. Therefore, for comparative purposes we selected two BciA homologues from *R. sphaeroides* and *C. tepidum* for BChl pathway extension based upon information gathered from sequence alignments.

**Figure 2:**
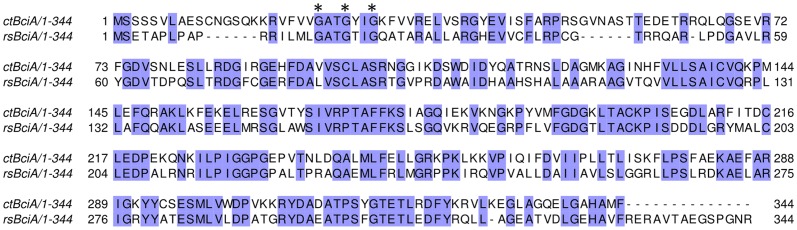
Amino acid sequence alignment of *Chlorobaculum tepidum CT*BciA and *Rhodobacter sphaeroides RS*BciA. The two divinyl reductases share 53% sequence identity. Conserved residues are highlighted in blue. The conserved GxxGxxG motif, required for NAD(P)H binding [48], is marked with asterisks.

The selected *R. sphaeroides* and *C. tepidum* BciA homologues are the most closely related of the previously characterized bacterial enzymes, sharing 53% sequence identity, and were therefore hypothesized to behave in a similar fashion. Additionally, both *RS*BciA and *CT*BciA have a well-defined GxxGxxG motif (Rossmann-fold, PF13460) [48] for binding of NAD(P)H. NADPH is essential for 8-vinyl reductase activity [27], [29] (Fig. 2). Attempts to model the 3D structure of the two full-length proteins using MODELLER v 9.12 [43] did not produce reliable results as no template structure with sufficient sequence similarity could be identified. However, the N-termini (residues 1–220) of both BciA homologues could be modeled using the Rossmann fold containing biliverdin IX β reductase as a template (PDB: 1HDO) [49] (Fig. S2), corroborating the expectation that both enzymes should carry out an NADPH-dependent reduction. Finally, we expected that the similarity of these genes to other components of our existing pathway, which includes members already derived from the closely related *R. capsulatus* and *C. tepidum*, would facilitate expression in *E. coli*.

### 8-vinyl Reductase Activity in *E. coli* Cells Expressing HemA-F, BchSID and BchM

For expression of the extended BChl pathway in *E. coli*, genes encoding *RS*BciA and *CT*BciA were cloned into our in house pCDFBB plasmid [37]. The BioBrick plasmid system has been designed for the straightforward stacking of several genes thereby facilitating pathway engineering, and constitutive expression is driven by a modified *lac* promoter. The pCDFBB-*ctbciA* and pCDFBB-*rsbciA* plasmids used in this study are compatible with the previously created pAC-*hemAD*, pBBR-*hemEF*, and pUCMOD-*SID* plasmids, allowing expression of the entire 11 gene pathway in a heterologous host. Additionally, by stacking *rsbciA* and *ctbciA* separately onto the pCDFBB-*bchM* plasmid, we maintained our original four plasmid system, reducing the likelihood of excessive metabolic burden placed on the cells due to different selective markers.

Using this plasmid system, we previously showed that P^IX^ overproducing *E. coli* expressing the Mg-chelatase BchSID complex produced MgP^IX^. We also showed that pathway extension with methyltransferase BchM resulted in the production of both MgP^IX^ME and the “dead-end” product P^IX^ME [15], findings which we also noted in this study (Table 1). However, in our current extended Bchl pathway, P^IX^ overproducing cells expressing BchSID and *CT*BciA alone produced two new compounds, mono-vinyl P^IX^ (mvP^IX^) and mono-vinyl MgP^IX^ (mvMgP^IX^), indicating that *CT*BciA was capable of reducing the C8-vinyl group on both the Mg chelated and the non-Mg chelated porphyrin molecule. Furthermore, upon expression of BchSID with BchM and *CT*BciA, MgP^IX^ME was no longer observed, with the concurrent appearance of the new product mono-vinyl MgP^IX^ME (mvMgP^IX^ME). While it is not known whether the methyltransferase BchM is producing less MgP^IX^ME when expressed in combination with the 8-vinyl reductase, it would appear that *CT*BciA catalyzes full conversion of the divinyl form of MgP^IX^ME to the mono-vinyl form. Additionally, levels of the “dead-end” product P^IX^ME were reduced upon coexpression with *CT*BciA, and mono-vinyl P^IX^ME (mvP^IX^ME) was produced. Notably, upon addition of *CT*BciA, there is a significant shift in the comparative levels of pathway intermediates derived from P^IX^ flowing towards dead-end non-Mg chelated products (P^IX^ME and mvP^IX^ME) or towards Mg-chelated products (MgP^IX^, MgP^IX^ME, mvMgP^IX^ and mvMgP^IX^ME). Without the 8-vinyl reductase, the BchSID-BchM extended pathway has a preference to produce non-Mg chelated products over Mg-chelated products at a ratio of 2∶1. With 8-vinyl reductase, this ratio is shifted to 1∶1, improving the pathway balance and resulting in more efficient use of the precursor P^IX^. Therefore, *CT*BciA can reduce the C8-vinyl group on a diverse range of intermediates in the Bchl pathway.

**Table 1:** Bacteriochlorophyll pathway intermediates produced by *E. coli* cells expressing various combinations of genes from the Heme and BChl pathways as detected by HPLC.

Gene combinations	Porphyrin products (mg L^−1^)							
	P^IX^	MgP^IX^	P^IX^ ME	MgP^IX^ ME	mvP^IX^	mvMgP^IX^	mvP^IX^ ME	mvMgP^IX^ ME
***HemA-F+BchSID***	80.9±17.2	45.8±7.5	ND	ND	ND	ND	ND	ND
***HemA-F+BchSID+BchM***	76.6±24.1	31.2±7	17.2±5	9.1±3.4	ND	ND	ND	ND
***HemA-F+BchSID+RSBciA***	90.2±2.2	28.4±4.6	ND	ND	ND	ND	ND	ND
***HemA-F+BchSID+CTBciA***	75.9±13.6	25.4±5.5	ND	ND	5.8±1.4	7.5±3.9	ND	ND
***HemA-*** ***F+BchSID+BchM*** **+** ***R*** ***SBciA***	74.7±25.4	37.6±2	14.6±2.6	9.5±1	ND	ND	ND	ND
***HemA-*** ***F+BchSID+BchM*** **+** ***C*** ***TBciA***	68.9±6.3	34.4±10.1	9.1±1.3	ND	4.3±0.4	5.2±3.1	2.9±0.2	3.4±0.6

*E. coli* cells expressing HemA-F and the magnesium chelatase complex BchSID produce P^IX^ and MgP^IX^. Addition of the methyl transferase BchM results in production of both P^IX^ ME and MgP^IX^ ME. Expression with the divinyl reductase *CT*BciA in the presence and absence of BchM leads to the production of mono-vinyl forms of pathway intermediates. *RS*BciA is not active in our *in vivo* system. Abbreviations: P^IX^ - protoporphyrin IX, MgP^IX^ - Mg-protoporphyrin IX, P^IX^ME - protoporphyrin IX methylester, MgP^IX^ME - Mg-protoporphyrin IX methylester, mvP^IX^ - mono-vinyl protoporphyrin IX, mvMgPIX - mono-vinyl Mg-protoporphyrin IX, mvP^IX^ME - mono-vinyl protoporphyrin IX methylester, mvMgP^IX^ME - mono-vinyl Mg-protoporphyrin IX methylester, ND – none detected.

Surprisingly, we found that *RS*BciA does not behave in a similar fashion to *CT*BciA. We did not detect mono-vinyl forms of any of the pathway intermediates upon coexpression of this 8-vinyl reductase with BchSID and BchM (Table 1), suggesting that *RS*BciA is either inactive in *E. coli* or does not display the same substrate promiscuity as *CT*BciA and instead specifically acts on divinyl protochlorophyllide (DVP) (refer to Fig. 1 for structures). This was an unexpected finding, as our BChl pathway design approach had previously been successful, enabling construction of pathways using enzymes from diverse bacterial sources [11], [15]. Our sequence analyses (the two proteins share 53% amino acid sequence identity and a conserved NAD(P)H binding motif) had given no prior indication that *RS*BciA would be different from *CT*BciA and not a suitable candidate for BChl pathway engineering.

We considered the possibility that BchJ, previously thought to play a role in the reduction of the C-8 vinyl group [50], may be an activator or otherwise facilitate expression of active *RS*BciA. To explore the potential activity of BchJ, we cloned *rsp_0280* from *R. sphaeroides* and expressed the protein (*RS*BchJ) in a similar manner to the BciA homologues. However, no mono-vinyl intermediates were observed in cultures expressing *RS*BchJ with BchSID and BchM. Previously, it had been reported that BchJ plays a substrate channeling role, rather than acting as a 8-vinyl reductase [27], therefore we hypothesized that BchJ could be involved in activating *RS*BciA. To test this possibility, we expressed *RS*BchJ in the presence of BchSID, BchM, and *RS*BciA. Once again, no 8-vinyl reductase activity was observed. Furthermore, coexpression of *RS*BchJ with *CT*BciA had no effect on 8-vinyl reductase activity. We therefore excluded BchJ from further analyses. To provide an explanation for the differences in activities of the two 8-vinyl reductase homologues, we set out to purify and characterize reduction of DVP and other P^IX^ derivatives by *RS*BciA and *CT*BciA *in vitro* to inform the design of current and future engineered pathways.

### Purification of the Two 8-vinyl Reductases

To understand the different activities observed for *RS*BciA and *CT*BciA in *E. coli*, we carried out the first purification of 8-vinyl reductases for detailed *in vitro* characterization. The genes encoding the homologues were cloned separately into the inducible plasmid pET30a(+) with a C-terminal His_6_ tag for purification purposes. The two enzymes were overexpressed in *E. coli* as soluble proteins, and were subsequently purified by metal-affinity chromatography upon elution from the column with 100–250 mM imidazole. Protein purity of *RS*BciA and *CT*BciA was estimated as >95% by SDS-PAGE analysis following a single purification step (Fig. S3).

Spectra for purified *RS*BciA and *CT*BciA revealed a maximum peak absorbance at 260 nm, suggesting that an unknown nucleotide(s) copurifies with the enzymes (Fig. S4). Attempts to determine its identity by mass spectrometry, however, were unsuccessful. Nonetheless, the conserved Rossmann fold NAD(P)H binding motif near the N-termini of the enzymes (Fig. 2) suggests binding of intercellular NAD(P)H shown for other NAD(P)H dependent proteins [51]. Binding of this nucleotide is sufficiently tight that it remains with the proteins following their elution as large complexes/aggregates from a size exclusion column (Fig. S5). *In vitro* assays with extracts of *E. coli* cells expressing *C. tepidum* BciA showed that the enzyme uses NADPH as co-factor in the reduction of divinyl protochlorophyllide (DVP) [27]. The presence of a putative tightly bound nucleotide following purification of *RS*BciA and *CT*BciA suggested that both purified proteins should be active *in vitro*.

### Reduction of Divinyl Protochlorophyllide by RSBciA and CTBciA

**Figure 3:**
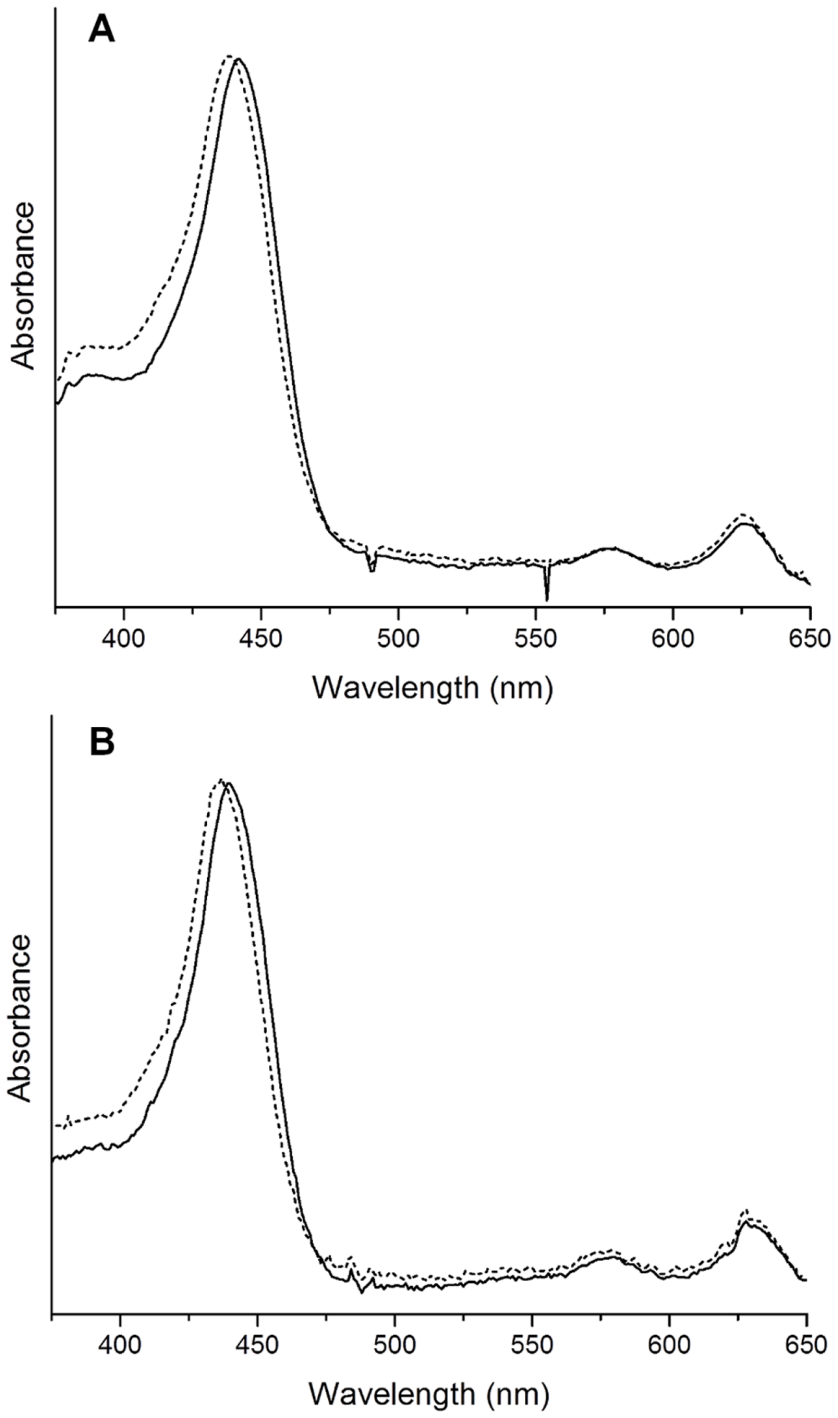
Changes in the absorbance spectrum of divinyl-protochlorophyllide upon addition of (A) *RS*BciA and (B) *CT*BciA. *In vitro* assays were carried out with NAD(P)H and with purified protein (**A**) *RS*BciA and (**B**) *CT*BciA. Divinyl-protochlorophyllide has a characteristic absorbance maximum of 442 nm (solid line). This shifts 5 nm to 337 nm upon the reduction of the C-8 vinyl group by the 8-vinyl reductase (dotted line) [27].

**Figure 4:**
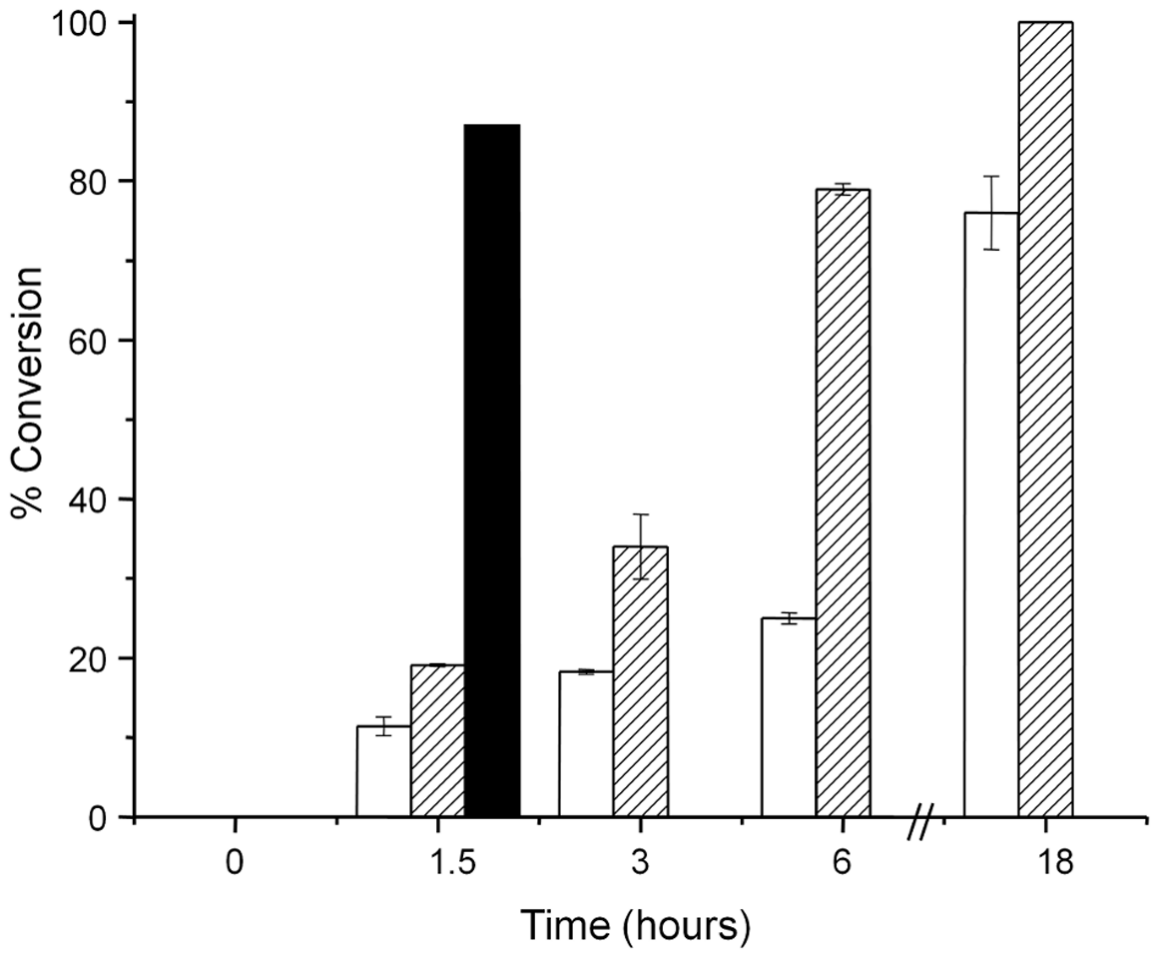
Reaction efficiency as a measure of percent conversion of divinyl-protochlorophyllide to mono-vinyl protochlorophyllide by 8-vinyl reductase. Purified *CT*BciA reduces greater than 85% DVP to mono-vinyl form in 1.5 hours (black bar). Purified *RS*BciA acts more slowly, reaching 100% conversion of divinyl to mono-vinyl in 18 hours (hashed bars). Attempts to improve reaction efficiency of *RS*BciA by addition of crude cell lysate to the reaction vessel actually reduced the rate of reaction as well as the overall conversion to less than 80% in 18 hours (white bars). Error bars are calculated from reactions carried out in duplicate.

To compare catalytic activities of *RS*BciA and *CT*BciA with divinyl protochlorophyllide (DVP), *in vitro* assays with purified protein supplemented with NADPH and DVP were conducted. For both *RS*BciA and *CT*BciA, DVP was reduced to the mono-vinyl form, resulting in the characteristic shift in absorbance maxima from 442 nm to 437 nm (Fig. 3) upon reduction of the C8-vinyl group [27], [28], [52]. Identity of the reaction products was further confirmed by LC-MS, revealing the addition of two protons to DVP upon reduction, with a shift in *m/z* from 610 to 612 (Fig. S6). These findings confirmed that *RS*BciA is active, albeit not *in vivo* with the engineered pathway. However, we did note a significant difference in the rate of reaction between the two enzymes, with the reaction containing *CT*BciA reaching almost 90% conversion of divinyl to mono-vinyl after 1.5 hours, and that of *RS*BciA with the same concentration of purified protein only reaching complete conversion after 18 hours (Fig. 4), despite numerous attempts at varying reaction conditions (eg. pH, temperature, buffer) Suspecting that *RS*BciA may require an additional cofactor or unknown chaperone, we supplemented the assay with *E. coli* crude cell lysate. Rather than improving reaction efficiency, adding cell lysate decreased the overall conversion of DVP to the mono-vinyl form by *RS*BciA from 100% to less than 80% after 18 hours, which could be attributed to an overall lower concentration of *RS*BciA in cell lysate, or to non-specific binding of NADPH and/or substrate to cell constituents.

It has recently been shown for plant 8-vinyl reductases that these enzymes have broad substrate specificities, with substrate preferences and activities varying according to species [36]. In some cases, 8-vinyl reductases are several hundred fold more efficient at converting a particular intermediate in chlorophyll biosynthesis than another [53]. As stated above, we considered the possibility that DVP is not the preferred substrate of *RS*BciA, and that it may be a more specific than *CT*BciA which acts in our engineered *E. coli* cells on any Mg-chelated or unchelated porphyrin IX derivative with a C8 divinyl group (Table 1).

### Substrate Promiscuity of *RS*BciA and *CT*BciA

**Figure 5:**
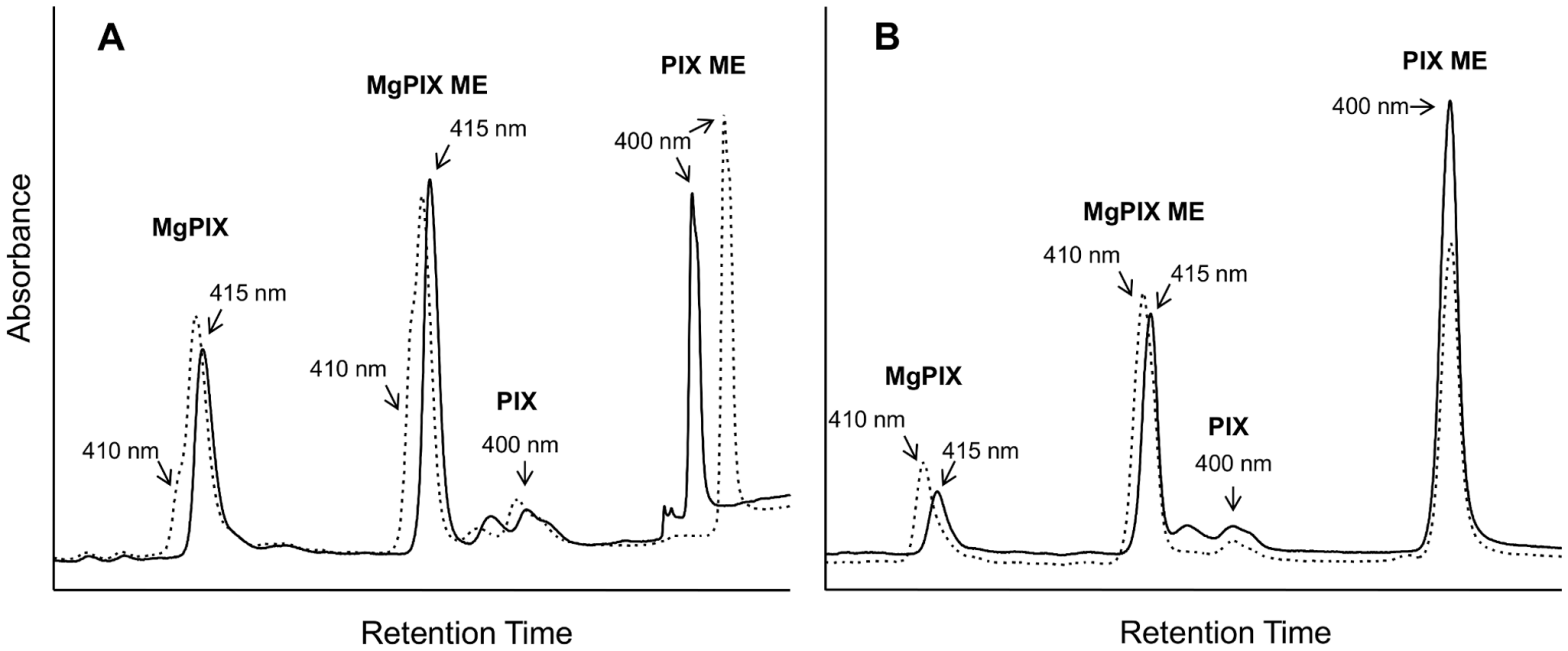
Substrate promiscuity of purified 8-vinyl reductases with BChl intermediates as determined by shifts in absorbance maxima. Conversion of a mixture of Bchl intermediates (MgP^IX^, MgP^IX^ME, P^IX^ME) was analyzed by HPLC at a single wavelength (412 nm) to detect all porphyrins present in the reaction mixtures after 18 hours. Reactions with enzyme (dotted traces) and control reactions (solid traces) are shown. Wavelengths displayed above arrows (pointing to peak shoulder or peak maximum) indicate the absorbance maximum measured at that time point, and illustrate the 5 nm absorbance shift which occurs after the reduction of the C-8 vinyl group. (**A**) Purified *RS*BciA partially reduces the C8-vinyl group of MgP^IX^ and MgP^IX^ME to generate a peak shoulder for each substrate at which the absorbance maximum is shifted from 415 nm to 410 nm [27]. Non-Mg chelated compounds are not reduced. Note that the shift in retention time observed for P^IX^ME in the enzyme and control reaction is the results from an aberrance in column running conditions as both compounds retain the absorbance maximum of the P^IX^ME substrate. (**B**) Purified *CT*BciA reduces the C8-vinyl group on MgP^IX^ and MgP^IX^ME, as indicated by a complete shift in compound peak absorbance maxima from 415 nm to 410 nm. No activity and correspondingly, no shift in absorbance maximum is observed against non-Mg chelated compounds P^IX^ and P^IX^ME. For abbreviations of substrate names see Table 1.

To determine the substrate preference of *RS*BciA, assays were carried out with purified protein in a reaction mixture containing the cell-extracted BChl pathway intermediates P^IX^, MgP^IX^, P^IX^ME and MgP^IX^ME, as well as reactions containing commercially available P^IX^ or MgP^IX^. In all cases, *RS*BciA reduced only the Mg-chelated porphyrins MgP^IX^ and MgP^IX^ME to the corresponding monovinyl products, no activity was observed with the unchelated P^IX^ derivatives (Fig. 5). Additionally, reaction rates were not improved over those obtained with DVP and full conversion of substrates was not observed, suggesting that substrate specificity is not the limiting factor for the inactivity of *RS*BciA in our pathway engineered *E. coli* cells.

Similar results were obtained for *CT*BciA, which also reduced only the Mg-chelated porphyrins at a similar rate to that observed for DVP (Fig. 5). Also, full conversion of Mg-chelated porphyrins was evident. These results indicate that both 8-vinyl reductases are substrate promiscuous, however activity is dependent upon the presence of the chelated magnesium. This is in contrast to data obtained from *in vivo* experiments, where *CT*BciA was apparently active against P^IX^ and P^IX^ME (Table 1). It could be that the presence of mvP^IX^ and mvP^IX^ME in our *in vivo* reactions is actually caused by loss of the magnesium ion during extraction of cell constituents, and that *CT*BciA is not truly active against the non-magnesium chelated porphyrins. Our *in vitro* data clearly show that *CT*BciA is not active against P^IX^ or P^IX^ME, which supports this theory.

The first committed step of BChl biosynthesis is the metal chelation of P^IX^ by the magnesium chelatase complex. Metal insertion is believed to tag porphyrin molecules for specific biosynthetic routes leading to e.g. hemes, BChls and corrins. BchHID gene disruptions in *R. capsulatus* result in accumulation of P^IX^, indicating that the metal ion is essential for the correct function of the downstream BChl enzymes [54], and studies in barley show that the presence of a metal ion is essential for reduction of protochlorophyllide [55]. It is believed that the metal ion is required for the correct orientation and binding of the porphyrin ring in the catalytic pocket of BChl pathway enzymes [56]. We therefore suspected that the difference in reaction rates between *RS*BciA and *CT*BciA may be related to different binding or interactions of the substrate(s) with the enzymes.

### Circular Dichroism Analysis of Substrate Binding

**Figure 6:**
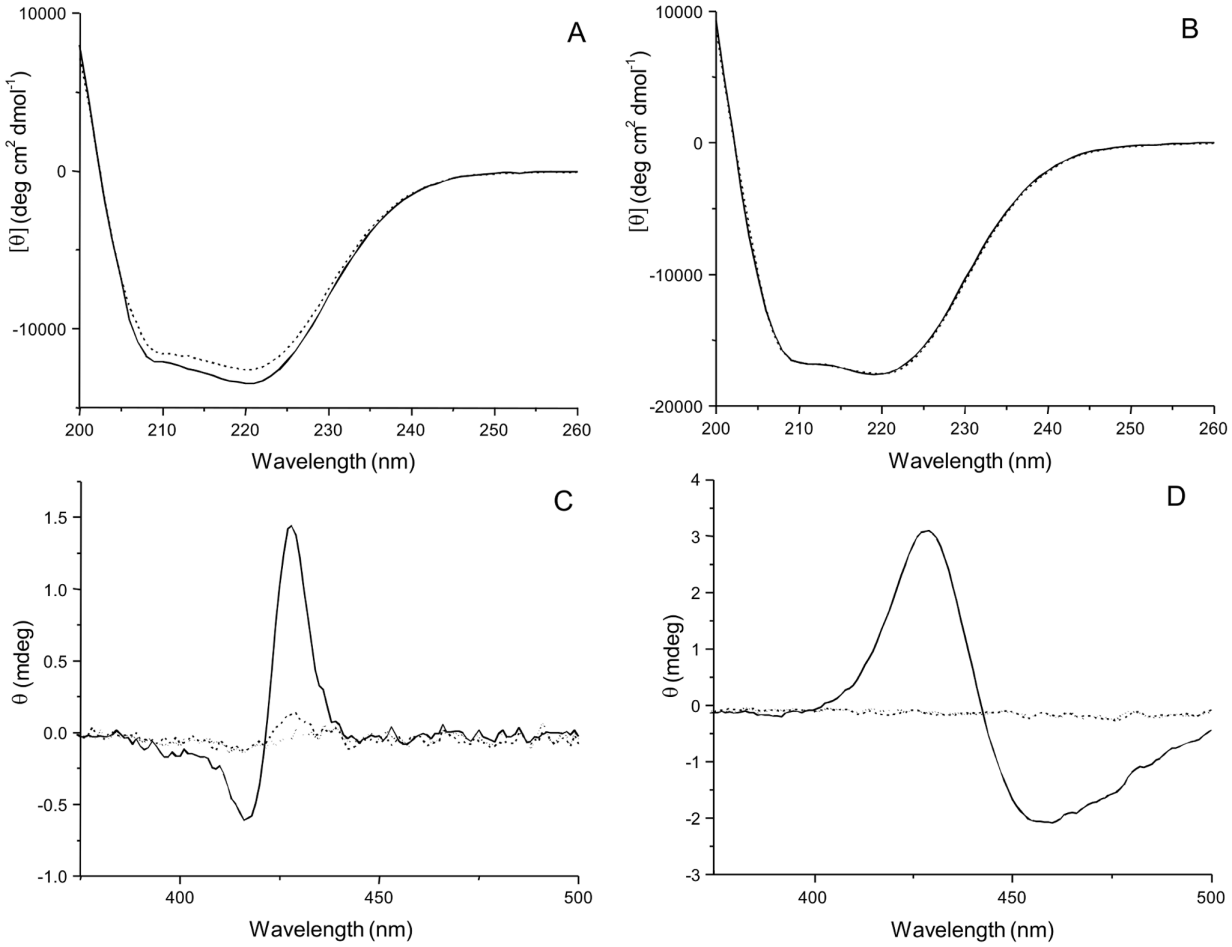
Circular dichroism analysis of the secondary structure of 8-vinyl reductase and the Soret band of MgP^IX^ reveals a difference in binding mode. CD spectra of purified protein in the far UV region show that (**A**) *CT*BciA and (**B**) *RS*BciA display the characteristic double minima at 222 nm and 208 nm associated with α-helical content [57] (solid line). Upon addition of MgP^IX^ to the protein, a shift is observed in the CD spectrum of *CT*BciA, but not *RS*BciA (dotted line). (**C**) and (**D**) Analysis in the Soret region of MgP^IX^ (dotted line) shows no spectra. Upon addition of purified protein (**C**) *CT*BciA and (**D**) *RS*BciA a change is observed in the Soret band of the porphyrin ring (solid line). The differences in peak and inflection wavelengths may represent MgP^IX^ interactions with different amino acid isomers in the two different proteins [58].

To understand the differences in rates of reaction between *RS*BciA and *CT*BciA, we sought to confirm whether the binding of substrate(s) was similar in the case of both 8-vinyl reductases using CD. Porphyrins and metallo-porphyrins are sensitive CD chromophores, ideal for detecting differences in substrate binding between the two proteins [57]. Wavescans in the far UV spectral region showed a change in the structure of *CT*BciA upon addition of MgP^IX^, with a shift in the characteristic double minima at 208 and 222 nm, associated with α-helical secondary structure [57] (Fig. 6A). This change was not observed upon addition of P^IX^ or ZnP^IX^, further confirming the dependence upon the chelated Mg^2+^ for correct binding of the substrate. Notably, there was no change in the structure of *RS*BciA upon addition of P^IX^, MgP^IX^, or ZnP^IX^ (Fig. 6B). However, changes in the Soret band of the MgP^IX^ chromophore were apparent upon addition of both *CT*BciA and *RS*BciA.

Studies into the binding of porphyrins by free amino acids indicate that the wavelength of the characteristic peak and inflection switches upon binding by D- or L-isomers of the same amino acid [58]. In our case, it could be that distinct side-chain conformational changes are taking place upon binding of MgP^IX^ in *CT*BciA and *RS*BciA, resulting in different changes in the Soret region (Fig. 6C, Fig. 6D) [58]. These data indicate that the two 8-vinyl reductases use a different substrate binding mode, which could be related to their differences in activity.

Interactions of Mg porphyrins with histidine, proline, serine, threonine and tryptophan result in a split CD signal [58]. Whether any of these residues are involved in hydrogen bonding or π stacking of the Mg-chelated porphyrins is not known in the case of 8-vinyl reductase, although it has been suggested that histidine plays a role in coordinating the chelated Mg^2+^ in other BChl pathway enzymes [56]. Which residue(s) is involved in coordinating the substrate is not immediately apparent by sequence analyses of *RS*BciA and *CT*BciA (Fig. 2), compounded by the fact that no model of the catalytic domain can be obtained (Fig. S2). Attempts to crystallize 8-vinyl reductase for 3D structure solution are currently underway, however diffraction quality crystals have not yet been obtained. Analysis of a crystal structure in the presence and absence of substrate could provide detailed information about the substrate binding mode and catalytic mechanism used by 8-vinyl reductase.

## Discussion

8-vinyl reductases are widely distributed in photosynthetic organisms. Putative 8-vinyl reductases have been identified in several different domains of life (Fig. S1), where they are suspected to catalyze a key functionalization of porphyrin molecules in (B)Chl biosynthesis [59]. Homologous enzymes exist across different species [36], as well as within individual species [60], highlighting the evolutionary selective advantage afforded by the catalytic role of 8-vinyl reductases. Recent studies revealed that homologous plant 8-vinyl reductases are substrate promiscuous, capable of reducing the C8-vinyl group of different pathway intermediates [36]. Data in this report demonstrates that substrate promiscuity is not only limited to plant 8-vinyl reductases, but is also a characteristic of 8-vinyl reductases from photosynthetic bacteria. Here, 8-vinyl reductases did not show a particular preference for any pathway intermediate, but activity was entirely dependent upon presence of the chelated Mg^2+^. These data are in agreement with previous studies, in which the presence of a chelated divalent metal ion was essential [54], [55].

It is not fully understood why the presence of a chelated Mg^2+^ in the porphyrin ring is essential for 8-vinyl reductase activity in this study. It is likely that the metal ion is required for the correct orientation of the porphyrin molecule in the active site or to sterically align the molecule in close proximity with the essential NADPH cofactor [56]. Our data provides some insights into the binding of the Mg-porphyrin substrate, which may affect molecular recognition and therefore catalytic activity. These data provide the first evidence that the mode of binding of substrate(s) varies between homologous 8-vinyl reductases. Whether this difference in binding confers a selective advantage to the host, or whether it provides the enzyme with a means to regulate reaction efficiency with differing substrates [36], and therefore pathway flux, remains open to question.

No data exists to show which residues are involved in binding the substrate or those which are involved in catalysis, which could explain the different modes of binding. Prior to this work, 8-vinyl reductase had not been purified, and limited biochemical data had been published [27], therefore the mechanistic details of the enzyme remained elusive. While this study has shed some light on the reduction of the C8-vinyl group of BChl intermediates by different 8-vinyl reductases, an in-depth analysis of the step-by-step catalytic process is still required. Detailed comparative biochemical and structural characterization of homologous 8-vinyl reductases would provide the information needed for a full understanding of the reaction mechanism and the substrate recognition of this enzyme.

The purpose of this study was to extend the engineered BChl biosynthetic pathway in *E. coli* [5]. It was necessary to insert a downstream enzyme that could accept the multiple products of our existing Mg chelatase-methyl transferase system [15]. In our endeavors to select a suitable candidate for this role, we discovered that *CT*BciA is capable of reducing the C8-vinyl group of several substrates when expressed as part of our engineered pathway. This is a significant step toward our goal of recreating the full BChl pathway in *E. coli*. One of the challenges of building a pathway engineered microbe is maintaining balance in pathway flux. Often, a slow catalytic rate of one or more enzymes can result in pathway bottlenecks, or unwanted side reactions can lead to inefficient use of metabolically expensive molecules [61]. We have not yet eliminated the potential for the production of non-Mg-chelated “dead end” porphyrins, although it is likely that the presence of mvP^IX^ME actually results from loss of the Mg ion from mvMgP^IX^ME upon extraction from the cell. Nonetheless, the addition of a substrate promiscuous 8-vinyl reductase to our system does provide a shuttle for the Mg-chelated intermediates, resulting in a reduction in the preference to produce P^IX^ME, and altering the pathway balance to produce equal ratios of the mono-vinyl Mg-chelated porphyrins (Table 1).

We were surprised to find that the closely related homologue *RS*BciA was inactive in our pathway. Furthermore, the *in vitro* catalytic rate of *RS*BciA was too slow to be relevant for our engineering purposes, despite extensive attempts to optimize reaction conditions. It may be that *RS*BciA requires an unknown species-specific cofactor, chaperone, interacting partner or shuttling enzyme for efficient function, which is the case for other enzymes involved in BChl biosynthesis [18], [62], [63]. It has been suggested by others that BchJ is not a 8-vinyl reductase as previously indicated [50], but that it functions as a carrier or shuttle for porphyrin intermediates [18]. However, we found that coexpression of *RS*BchJ had no effect on *RS*BciA activity. Future studies beyond gene-knockouts could elucidate the exact nature of BciA behavior in *R. sphaeroides*, and could clarify whether this particular 8-vinyl reductase is capable of acting alone or whether its activity is upregulated in the presence of certain other members of the pathway or under different reaction conditions [16]. Very recently, an anaerobic 8-vinyl reductase (BciB) from the green sulfur bacterium *Chloroherpeton thalassium* was characterized. BciB requires two [4Fe-4S] clusters, FAD, and a reductant such as ferredoxin or sodium dithionite to reduce the C-8 vinyl group of DVP [64]. This study reveals the mechanistic diversity of 8-vinyl reductases, and highlights the importance of *in vitro* characterization for a full appreciation of optimal conditions for catalysis. Furthermore, the presence of a not-yet-identified 8-vinyl reductase in *R. sphaeroides* cannot be ruled out, as we are only just gaining insights into the sequence diversity of this class of enzymes [60].

Relying upon sequence analyses and the relatively limited biochemical data that existed for 8-vinyl reductases was not sufficient for the strategic design of our engineered BChl system in *E. coli*. We were not able to predict that *CT*BciA would be active in our engineered pathway and that *RS*BciA would be inactive in the same engineered pathway. The unsuitability of *RS*BciA for our purposes only became truly apparent upon our own detailed biochemical and biophysical characterization of the two enzymes. In some ways, this study serves as a good example to underline the fact that the strategic and streamlined design and engineering of metabolic pathways is heavily dependent upon having a detailed knowledge of the catalytic mechanism and/or three dimensional structure of the enzyme(s) in question [65]. Sometimes this data is not available to the pathway engineer, in which case it becomes necessary to characterize the enzymes involved, and gather the detailed information needed to optimize the system for a particular purpose. Pathway engineering is not a straightforward process of building a chain of enzymes to make a product, rather it is the intricate design, creation and polishing of a living system to entice it to carry out a completely new activity.

In conclusion, this study provides an in-depth characterization of two 8-vinyl reductases from two photosynthetic organisms, and gives insights into the potential diversity of function with regards to substrate promiscuity and binding of substrates. This study brings us a step closer to the realization of the creation of an industrially relevant synthetic microbe that can use sunlight as a cheap source of energy to drive the biosynthesis of valuable and designer target molecules.

## Supporting Information

Figure S1 **Phylogenetic analyses of 8-vinyl reductases to select candidates for pathway engineering.** BLAST searches using *RS*BciA as search template identify 37 putative 8-vinyl reductases that share greater than 30% sequence identity. Homologues cluster with other members of the various domains of life, highlighted by colored boxes. Note that the cyanobacteria *Acaryochloris marina* MBIC11017 and *Leptolyngbya* sp. PCC 7376 appear to have obtained copies of 8-vinyl reductase by a process of lateral gene transfer from the α-proteobacteria. Five of the identified 8-vinyl reductases have been previously characterized, highlighted with asterisks. BciA from *R. sphaeroides* and *Chlorobaculum* (*Chlorobium*) *tepidum*, marked with arrows, are the two bacterial characterized 8-vinyl reductases that were selected to extend our engineered BChl pathway in *E. coli*.
(TIF)

Figure S2 **Structural models of the N-terminal domains of** ***RS*** **BciA and** ***CT*** **BciA. (A)**. Human biliverdin IX β reductase (1HDO), which has a characteristic Rossmann fold and binds NADP [49], was used to create structural models of the N-terminal domain of 8-vinyl reductases: **(B)** *RS*BciA residues 12–218 and **(C)** *CT*BciA residues 16–231. The conserved NAD(P)H binding motif GxxGxxG [48] is highlighted in red. The C-terminal domains of the divinyl reductases could not be modeled because no suitable X-ray structure as modeling template exists.
(TIF)

Figure S3 **Metal-affinity purification of recombinant** ***RS*** **BciA and** ***CT*** **BciA expressed in** ***E. coli*** **. (A)** SDS-PAGE analysis of *RS*BciA purification. M indicates the molecular weight marker with the corresponding weight (kDa) labeled on the left. Lane 1 shows the soluble portion of the cell lysate. Lane 2 shows contaminating proteins that did not bind to the Ni^2+^ affinity column, as well as excess *RS*BciA. Lanes 3–9 show the elution of pure ∼37 kDa *RS*BciA from the column in concentrations 85–220 mM imidazole. **(B)** SDS-PAGE analysis of *CT*BciA purification. M indicates the molecular weight marker with the corresponding weight (kDa) labeled on the left. Lanes 1 and 2 shows the total and soluble portion of the cell lysate. Lane 3 shows contaminating proteins that did not bind to the Ni^2+^ affinity column, as well as excess *CT*BciA. Lanes 4–7 show elution from the column with increasing concentrations of imidazole. Lane 8 shows elution of pure *CT*BciA in 250 mM imidazole.
(TIF)

Figure S4 **Nucleotide(s) copurify with recombinant** ***RS*** **BciA and** ***CT*** **BciA.** UV/Vis wavescans of purified 8-vinyl reductases *RS*BciA **(A)** and *CT*BciA **(B)** reveal absorbance maxima at 260 nm. This suggests that an unknown nucleotide(s) co-purifies with the enzymes, likely due to the presence of the conserved and essential NADPH binding site at the N-termini of both proteins.
(TIF)

Figure S5 **Recombinant 8-vinyl reductases behave as large complexes/aggregates in solution. (A)**. *RS*BciA elutes from a size exclusion column after 8.5 mL, close to the void volume of the column. RSBciA appears to be aggregating in solution, despite the presence of the protein stabilizing agent glycerol (10%). **(B)**. *CT*BciA elutes from the same size exclusion column after 10 mL, suggesting that it is forming a large complex close to 600 kDa (as determined using protein standards of known molecular weight).
(TIF)

Figure S6 **Mass spectra of divinyl-protochlorophyllide and mono-vinyl-protochlorophyllide.** **(A)**. The peak at *m/z* 610 is characteristic of divinyl-protochlorophyllide. **(B)**. Upon the reduction of divinyl-protochlorophyllide by *RS*BciA and *CT*BciA to the mono-vinyl form, two protons are added and the mass shifts to *m/z* 612. The peaks at *m/z* 642 and 644, respectively, represent methanol adducts of the two compounds.
(TIF)

Table S1 **Primers used in this study.** Restriction sites are represented in lower case.
(DOCX)

Table S2 **Plasmids used in this study.**
(DOCX)

## References

[1] Rosgaard L, de Porcellinis AJ, Jacobsen JH, Frigaard NU, Sakuragi Y (2012) Bioengineering of carbon fixation, biofuels, and biochemicals in cyanobacteria and plants. J Biotechnol 162: 134–147. 22677697 10.1016/j.jbiotec.2012.05.006

[2] Machado IM, Atsumi S (2012) Cyanobacterial biofuel production. J Biotechnol 162: 50–56. 22446641 10.1016/j.jbiotec.2012.03.005

[3] Work VH, D'Adamo S, Radakovits R, Jinkerson RE, Posewitz MC (2012) Improving photosynthesis and metabolic networks for the competitive production of phototroph-derived biofuels. Curr Opin Biotechnol 23: 290–297. 22172528 10.1016/j.copbio.2011.11.022

[4] Maurino VG, Weber AP (2013) Engineering photosynthesis in plants and synthetic microorganisms. J Exp Bot 64: 743–751. 23028016 10.1093/jxb/ers263

[5] Johnson ET, Schmidt-Dannert C (2008) Light-energy conversion in engineered microorganisms. Trends Biotechnol 26: 682–689. 18951642 10.1016/j.tibtech.2008.09.002

[6] Tikh I, Schmidt-Dannert C (2013) Towards Engineered Light-Energy Conversion in Nonphotosynthetic Microorganisms. Synthetic Biology: Tools and Applications. 303–316.

[7] Weeks AM, Chang MC (2011) Constructing *de novo* biosynthetic pathways for chemical synthesis inside living cells. Biochemistry 50: 5404–5418. 21591680 10.1021/bi200416g PMC3768262

[8] Johnson ET, Baron DB, Naranjo B, Bond DR, Schmidt-Dannert C, et al (2010) Enhancement of survival and electricity production in an engineered bacterium by light-driven proton pumping. Appl Environ Microbiol 76: 4123–4129. 20453141 10.1128/AEM.02425-09 PMC2897463

[9] Mijts BN, Lee PC, Schmidt-Dannert C (2005) Identification of a carotenoid oxygenase synthesizing acyclic xanthophylls: combinatorial biosynthesis and directed evolution. Chem Biol 12: 453–460. 15850982 10.1016/j.chembiol.2005.02.010

[10] Mijts BN, Lee PC, Schmidt-Dannert C (2004) Engineering carotenoid biosynthetic pathways. Methods Enzymol 388: 315–329. 15289080 10.1016/S0076-6879(04)88025-X

[11] Kwon SJ, de Boer AL, Petri R, Schmidt-Dannert C (2003) High-level production of porphyrins in metabolically engineered *Escherichia coli*: systematic extension of a pathway assembled from overexpressed genes involved in heme biosynthesis. Appl Environ Microbiol 69: 4875–4883. 12902282 10.1128/AEM.69.8.4875-4883.2003 PMC169110

[12] Battersby AR, Fookes CJ, Matcham GW, McDonald E (1980) Biosynthesis of the pigments of life: formation of the macrocycle. Nature 285: 17–21. 6769048 10.1038/285017a0

[13] Chew AG, Bryant DA (2007) Chlorophyll biosynthesis in bacteria: the origins of structural and functional diversity. Annu Rev Microbiol 61: 113–129. 17506685 10.1146/annurev.micro.61.080706.093242

[14] Willows RD (2003) Biosynthesis of chlorophylls from protoporphyrin IX. Nat Prod Rep 20: 327–341. 12828371 10.1039/b110549n

[15] Johnson ET, Schmidt-Dannert C (2008) Characterization of three homologs of the large subunit of the magnesium chelatase from *Chlorobaculum tepidum* and interaction with the magnesium protoporphyrin IX methyltransferase. J Biol Chem 283: 27776–27784. 18693239 10.1074/jbc.M804486200

[16] Bollivar DW, Jiang ZY, Bauer CE, Beale SI (1994) Heterologous expression of the *bchM* gene product from *Rhodobacter capsulatus* and demonstration that it encodes S-adenosyl-L-methionine:Mg-protoporphyrin IX methyltransferase. J Bacteriol 176: 5290–5296. 8071204 10.1128/jb.176.17.5290-5296.1994 PMC196713

[17] Ouchane S, Steunou AS, Picaud M, Astier C (2004) Aerobic and anaerobic Mg-protoporphyrin monomethyl ester cyclases in purple bacteria: a strategy adopted to bypass the repressive oxygen control system. J Biol Chem 279: 6385–6394. 14617630 10.1074/jbc.M309851200

[18] Sawicki A, Willows RD (2010) BchJ and BchM interact in a 1 : 1 ratio with the magnesium chelatase BchH subunit of *Rhodobacter capsulatus* . FEBS J 277: 4709–4721. 20955518 10.1111/j.1742-4658.2010.07877.x

[19] Boldareva-Nuianzina EN, Blahova Z, Sobotka R, Koblizek M (2013) Distribution and origin of oxygen-dependent and oxygen-independent forms of Mg-protoporphyrin monomethylester cyclase among phototrophic proteobacteria. Appl Environ Microbiol 79: 2596–2604. 23396335 10.1128/AEM.00104-13 PMC3623192

[20] Tang KH, Wen J, Li X, Blankenship RE (2009) Role of the AcsF protein in Chloroflexus aurantiacus. J Bacteriol 191: 3580–3587. 19346304 10.1128/JB.00110-09 PMC2681904

[21] Fujita Y, Matsumoto H, Takahashi Y, Matsubara H (1993) Identification of a nifDK-like gene (*ORF467*) involved in the biosynthesis of chlorophyll in the cyanobacterium *Plectonema boryanum* . Plant Cell Physiol 34: 305–314. 8199775

[22] Schulz R, Steinmuller K, Klaas M, Forreiter C, Rasmussen S, et al (1989) Nucleotide sequence of a cDNA coding for the NADPH-protochlorophyllide oxidoreductase (PCR) of barley (*Hordeum vulgare* L.) and its expression in *Escherichia coli* . Mol Gen Genet 217: 355–361. 2671659 10.1007/BF02464904

[23] Brocker MJ, Schomburg S, Heinz DW, Jahn D, Schubert WD, et al (2010) Crystal structure of the nitrogenase-like dark operative protochlorophyllide oxidoreductase catalytic complex (ChlN/ChlB)2. J Biol Chem 285: 27336–27345. 20558746 10.1074/jbc.M110.126698 PMC2930732

[24] Moser J, Lange C, Krausze J, Rebelein J, Schubert WD, et al (2013) Structure of ADP-aluminium fluoride-stabilized protochlorophyllide oxidoreductase complex. Proc Natl Acad Sci U S A 110: 2094–2098. 23341615 10.1073/pnas.1218303110 PMC3568340

[25] Muraki N, Nomata J, Ebata K, Mizoguchi T, Shiba T, et al (2010) X-ray crystal structure of the light-independent protochlorophyllide reductase. Nature 465: 110–114. 20400946 10.1038/nature08950

[26] Paddock T, Lima D, Mason ME, Apel K, Armstrong GA (2012) Arabidopsis light-dependent protochlorophyllide oxidoreductase A (PORA) is essential for normal plant growth and development. Plant Mol Biol 78: 447–460. 22278767 10.1007/s11103-012-9873-6

[27] Chew AG, Bryant DA (2007) Characterization of a plant-like protochlorophyllide *a* divinyl reductase in green sulfur bacteria. J Biol Chem 282: 2967–2975. 17148453 10.1074/jbc.M609730200

[28] Nagata N, Tanaka R, Satoh S, Tanaka A (2005) Identification of a vinyl reductase gene for chlorophyll synthesis in *Arabidopsis thaliana* and implications for the evolution of *Prochlorococcus* species. Plant Cell 17: 233–240. 15632054 10.1105/tpc.104.027276 PMC544501

[29] Parham R, Rebeiz CA (1992) Chloroplast biogenesis: [4-vinyl] chlorophyllide *a* reductase is a divinyl chlorophyllide *a*-specific, NADPH-dependent enzyme. Biochemistry 31: 8460–8464. 1390630 10.1021/bi00151a011

[30] Canniffe DP, Jackson PJ, Hollingshead S, Dickman MJ, Hunter CN (2013) Identification of an 8-vinyl reductase involved in bacteriochlorophyll biosynthesis in *Rhodobacter sphaeroides* and evidence for the existence of a third distinct class of the enzyme. Biochem J 450: 397–405. 23252506 10.1042/BJ20121723

[31] Sytina OA, Heyes DJ, Hunter CN, Groot ML (2009) Ultrafast catalytic processes and conformational changes in the light-driven enzyme protochlorophyllide oxidoreductase (POR). Biochem Soc Trans 37: 387–391. 19290868 10.1042/BST0370387

[32] Eckhardt U, Grimm B, Hortensteiner S (2004) Recent advances in chlorophyll biosynthesis and breakdown in higher plants. Plant Mol Biol 56: 1–14. 15604725 10.1007/s11103-004-2331-3

[33] Masuda T (2008) Recent overview of the Mg branch of the tetrapyrrole biosynthesis leading to chlorophylls. Photosynth Res 96: 121–143. 18273690 10.1007/s11120-008-9291-4

[34] Shisler KA, Broderick JB (2012) Emerging themes in radical SAM chemistry. Curr Opin Struct Biol 22: 701–710. 23141873 10.1016/j.sbi.2012.10.005 PMC4083504

[35] Nagata N, Tanaka R, Tanaka A (2007) The major route for chlorophyll synthesis includes [3,8-divinyl]-chlorophyllide *a* reduction in *Arabidopsis thaliana* . Plant Cell Physiol 48: 1803–1808. 17991629 10.1093/pcp/pcm153

[36] Wang P, Wan C, Xu Z, Wang P, Wang W, et al (2013) One divinyl reductase reduces the 8-vinyl groups in various intermediates of chlorophyll biosynthesis in a given higher plant species, but the isozyme differs between species. Plant Physiol 161: 521–534. 23154534 10.1104/pp.112.208421 PMC3532282

[37] Vick JE, Johnson ET, Choudhary S, Bloch SE, Lopez-Gallego F, et al (2011) Optimized compatible set of BioBrick vectors for metabolic pathway engineering. Appl Microbiol Biotechnol 92: 1275–1286. 22033566 10.1007/s00253-011-3633-4

[38] Tamura K, Peterson D, Peterson N, Stecher G, Nei M, et al (2011) MEGA5: molecular evolutionary genetics analysis using maximum likelihood, evolutionary distance, and maximum parsimony methods. Mol Biol Evol 28: 2731–2739. 21546353 10.1093/molbev/msr121 PMC3203626

[39] Thompson JD, Gibson TJ, Higgins DG (2002) Multiple sequence alignment using ClustalW and ClustalX. Curr Protoc Bioinformatics Chapter 2: Unit 2 3. 10.1002/0471250953.bi0203s00 18792934

[40] Altschul SF, Gish W, Miller W, Myers EW, Lipman DJ (1990) Basic local alignment search tool. J Mol Biol 215: 403–410. 2231712 10.1016/S0022-2836(05)80360-2

[41] Saitou N, Nei M (1987) The neighbor-joining method: a new method for reconstructing phylogenetic trees. Mol Biol Evol 4: 406–425. 3447015 10.1093/oxfordjournals.molbev.a040454

[42] Felsenstein J (1992) Estimating effective population size from samples of sequences: a bootstrap Monte Carlo integration method. Genet Res 60: 209–220. 1286805 10.1017/s0016672300030962

[43] Eswar N, Webb B, Marti-Renom MA, Madhusudhan MS, Eramian D, et al.. (2006) Comparative protein structure modeling using Modeller. Curr Protoc Bioinformatics Chapter 5: Unit 5 6. 10.1002/0471250953.bi0506s15 PMC4186674 18428767

[44] Sreerama N, Woody RW (2000) Estimation of protein secondary structure from circular dichroism spectra: comparison of CONTIN, SELCON, and CDSSTR methods with an expanded reference set. Anal Biochem 287: 252–260. 11112271 10.1006/abio.2000.4880

[45] Mackenzie C, Eraso JM, Choudhary M, Roh JH, Zeng X, et al (2007) Postgenomic adventures with *Rhodobacter sphaeroides* . Annu Rev Microbiol 61: 283–307. 17506668 10.1146/annurev.micro.61.080706.093402

[46] Mizoguchi T, Harada J, Tamiaki H (2012) Characterization of chlorophyll pigments in the mutant lacking 8-vinyl reductase of green photosynthetic bacterium *Chlorobaculum tepidum* . Bioorg Med Chem 20: 6803–6810. 23098608 10.1016/j.bmc.2012.09.054

[47] Shen F (2008) Purification and kinetic characterization of protochlorophyllide a divinyl reductase in green sulfur bacterium *Chlorobium tepidum* M Sc Thesis.

[48] Rescigno M, Perham RN (1994) Structure of the NADPH-binding motif of glutathione reductase: efficiency determined by evolution. Biochemistry 33: 5721–5727. 8180198 10.1021/bi00185a008

[49] Pereira PJ, Macedo-Ribeiro S, Parraga A, Perez-Luque R, Cunningham O, et al (2001) Structure of human biliverdin IXβ reductase, an early fetal bilirubin IXβ producing enzyme. Nat Struct Biol 8: 215–220. 11224564 10.1038/84948

[50] Suzuki JY, Bauer CE (1995) Altered monovinyl and divinyl protochlorophyllide pools in bchJ mutants of *Rhodobacter capsulatus*. Possible monovinyl substrate discrimination of light-independent protochlorophyllide reductase. J Biol Chem 270: 3732–3740. 7876113

[51] Kim MH, Kim Y, Park HJ, Lee JS, Kwak SN, et al (2008) Structural insight into bioremediation of triphenylmethane dyes by *Citrobacter* sp. triphenylmethane reductase. J Biol Chem 283: 31981–31990. 18782772 10.1074/jbc.M804092200

[52] Kolossov VL, Rebeiz CA (2001) Chloroplast biogenesis 84: solubilization and partial purification of membrane-bound [4-vinyl]chlorophyllide *a* reductase from etiolated barley leaves. Anal Biochem 295: 214–219. 11488624 10.1006/abio.2001.5195

[53] Parham R, Rebeiz CA (1995) Chloroplast biogenesis 72: a [4-vinyl]chlorophyllide *a* reductase assay using divinyl chlorophyllide *a* as an exogenous substrate. Anal Biochem 231: 164–169. 8678296 10.1006/abio.1995.1516

[54] Bollivar DW, Suzuki JY, Beatty JT, Dobrowolski JM, Bauer CE (1994) Directed mutational analysis of bacteriochlorophyll *a* biosynthesis in *Rhodobacter capsulatus* . J Mol Biol 237: 622–640. 8158642 10.1006/jmbi.1994.1260

[55] Griffiths WT (1980) Substrate-specificity studies on protochlorophyllide reductase in barley (*Hordeum vulgare*) etioplast membranes. Biochem J 186: 267–278. 7370013 10.1042/bj1860267 PMC1161527

[56] Townley HE, Sessions RB, Clarke AR, Dafforn TR, Griffiths WT (2001) Protochlorophyllide oxidoreductase: a homology model examined by site-directed mutagenesis. Proteins 44: 329–335. 11455606 10.1002/prot.1098

[57] Greenfield N, Fasman GD (1969) Computed circular dichroism spectra for the evaluation of protein conformation. Biochemistry 8: 4108–4116. 5346390 10.1021/bi00838a031

[58] Huang X, Nakanishi K, Berova N (2000) Porphyrins and metalloporphyrins: versatile circular dichroic reporter groups for structural studies. Chirality 12: 237–255. 10790194 10.1002/(SICI)1520-636X(2000)12:4<237::AID-CHIR10>3.0.CO;2-6

[59] Senge MO (2011) Stirring the porphyrin alphabet soup–functionalization reactions for porphyrins. Chem Commun (Camb) 47: 1943–1960. 21218237 10.1039/c0cc03984e

[60] Liu Z, Bryant DA (2011) Multiple types of 8-vinyl reductases for (bacterio)chlorophyll biosynthesis occur in many green sulfur bacteria. J Bacteriol 193: 4996–4998. 21764919 10.1128/JB.05520-11 PMC3165659

[61] Lopez-Gallego F, Schmidt-Dannert C (2010) Multi-enzymatic synthesis. Curr Opin Chem Biol 14: 174–183. 20036183 10.1016/j.cbpa.2009.11.023

[62] Hinchigeri SB, Hundle B, Richards WR (1997) Demonstration that the BchH protein of *Rhodobacter capsulatus* activates S-adenosyl-L-methionine:magnesium protoporphyrin IX methyltransferase. FEBS Lett 407: 337–342. 9175880 10.1016/s0014-5793(97)00371-2

[63] Willows RD, Gibson LC, Kanangara CG, Hunter CN, von Wettstein D (1996) Three separate proteins constitute the magnesium chelatase of *Rhodobacter sphaeroides* . Eur J Biochem 235: 438–443. 8631364 10.1111/j.1432-1033.1996.00438.x

[64] Saunders AH, Golbeck JH, Bryant DA (2013) Characterization of BciB, a ferredoxin-dependent 8-vinyl-protochlorophyllide reductase from the green sulfur bacterium Chloroherpeton thalassium. Biochemistry. 10.1021/bi401172b 24151992

[65] Mijts BN, Schmidt-Dannert C (2003) Engineering of secondary metabolite pathways. Curr Opin Biotechnol 14: 597–602. 14662388 10.1016/j.copbio.2003.09.009

