# Correction: A Tale of Two Reductases: Extending the Bacteriochlorophyll Biosynthetic Pathway in *E. coli*

**DOI:** 10.1371/journal.pone.0093807

**Published:** 2014-03-25

**Authors:** The PLOS ONE Staff

**Notice of Republication**

This article was republished on March 11, 2014, due to incorrect figure order. Please download this article again to view the correct version. The originally published, uncorrected article and the republished, corrected article are provided here for reference.

## Supporting Information

File S1 Originally published, uncorrected article.
(PDF)

File S2 Republished, corrected article.
(PDF)
