# Supplementary figures and images for: A Tale of Two Reductases: Extending the Bacteriochlorophyll Biosynthetic Pathway in E. coli
Source: PLoS One. 2014 Feb 21;9(2):e89734. doi: 10.1371/journal.pone.0089734 (PMC3931815; doi:10.1371/journal.pone.0089734)

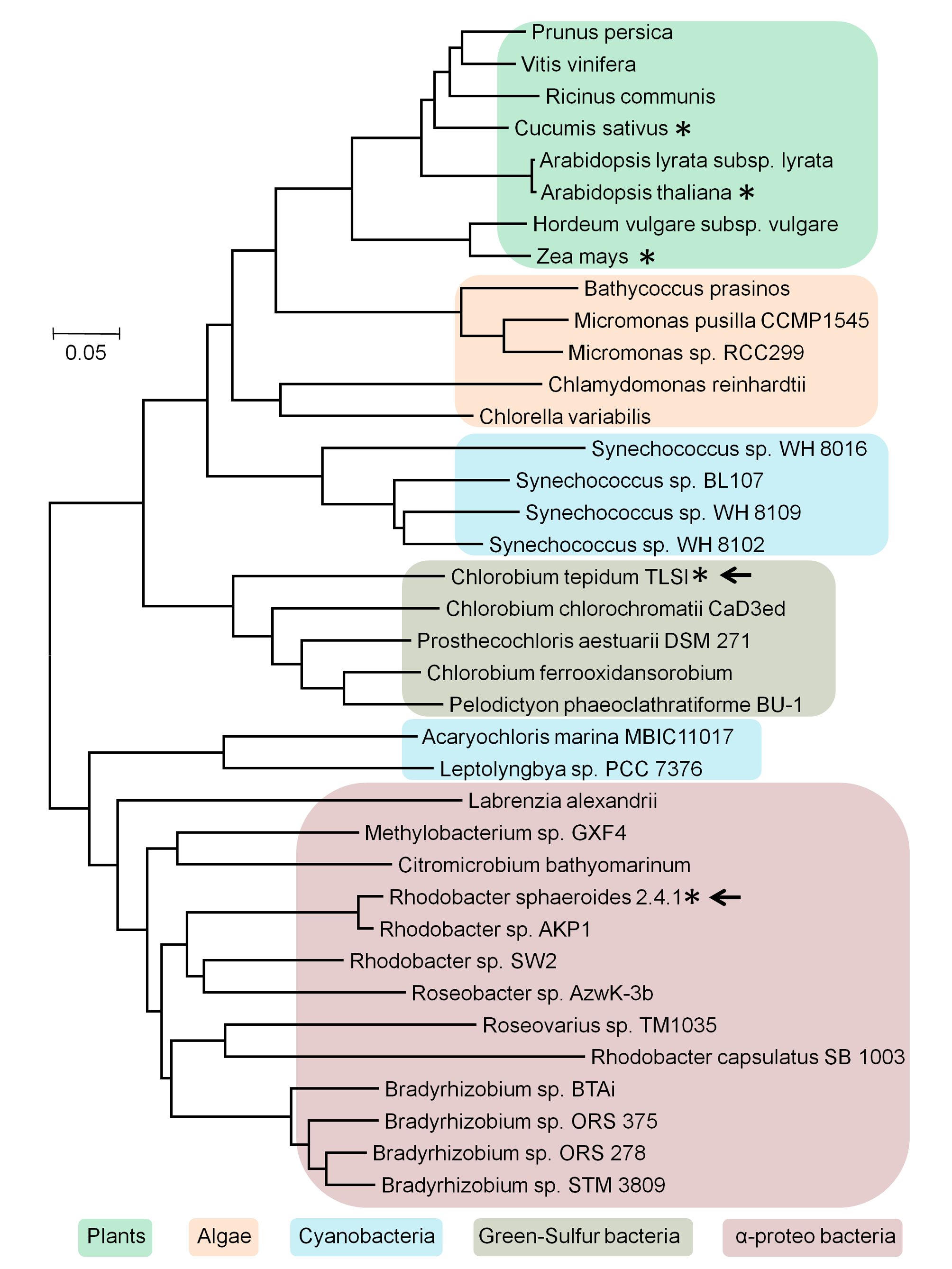

Supplement: Figure S1 — Phylogenetic analyses of 8-vinyl reductases to select candidates for pathway engineering. BLAST searches using RSBciA as search template identify 37 putative 8-vinyl reductases that share greater than 30% sequence identity. Homologues cluster with other members of the various domains of life, highlighted by colored boxes. Note that the cyanobacteria Acaryochloris marina MBIC11017 and Leptolyngbya sp. PCC 7376 appear to have obtained copies of 8-vinyl reductase by a process of lateral gene transfer from the α-proteobacteria. Five of the identified 8-vinyl reductases have been previously characterized, highlighted with asterisks. BciA from R. sphaeroides and Chlorobaculum (Chlorobium) tepidum, marked with arrows, are the two bacterial characterized 8-vinyl reductases that were selected to extend our engineered BChl pathway in E. coli. (TIF) [file pone.0089734.s001.tif]

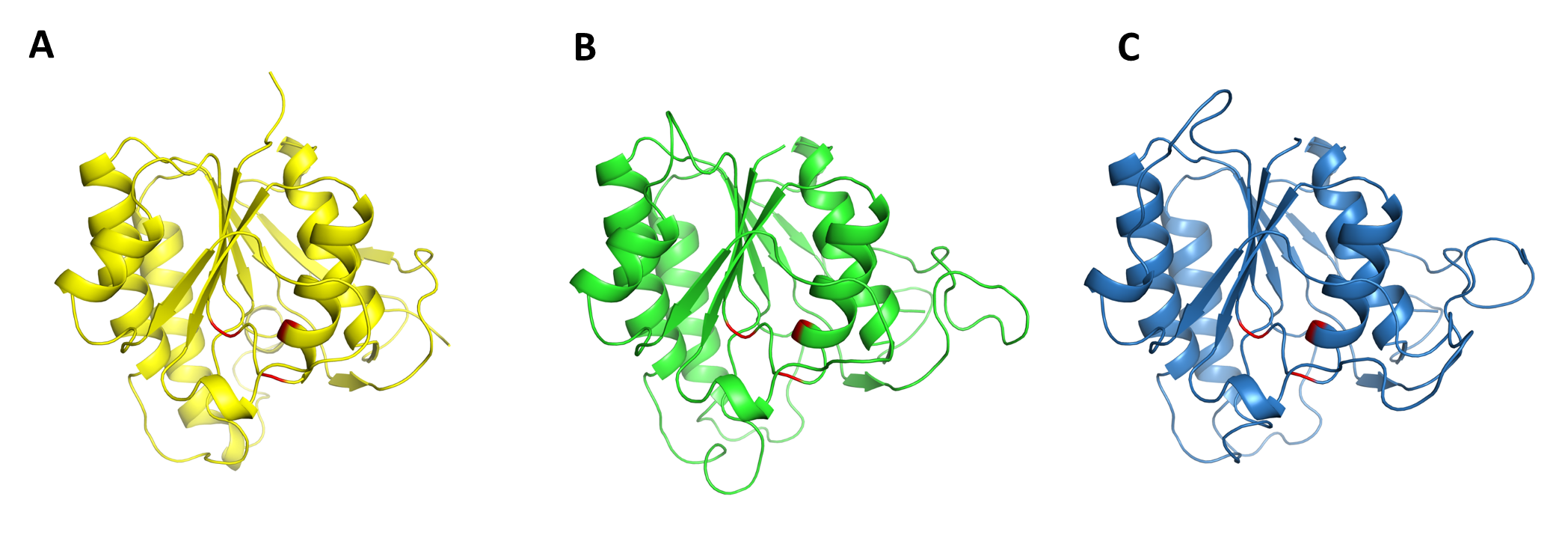

Supplement: Figure S2 — Structural models of the N-terminal domains of RS BciA and CT BciA. (A). Human biliverdin IX β reductase (1HDO), which has a characteristic Rossmann fold and binds NADP [49], was used to create structural models of the N-terminal domain of 8-vinyl reductases: (B) RSBciA residues 12–218 and (C) CTBciA residues 16–231. The conserved NAD(P)H binding motif GxxGxxG [48] is highlighted in red. The C-terminal domains of the divinyl reductases could not be modeled because no suitable X-ray structure as modeling template exists. (TIF) [file pone.0089734.s002.tif]

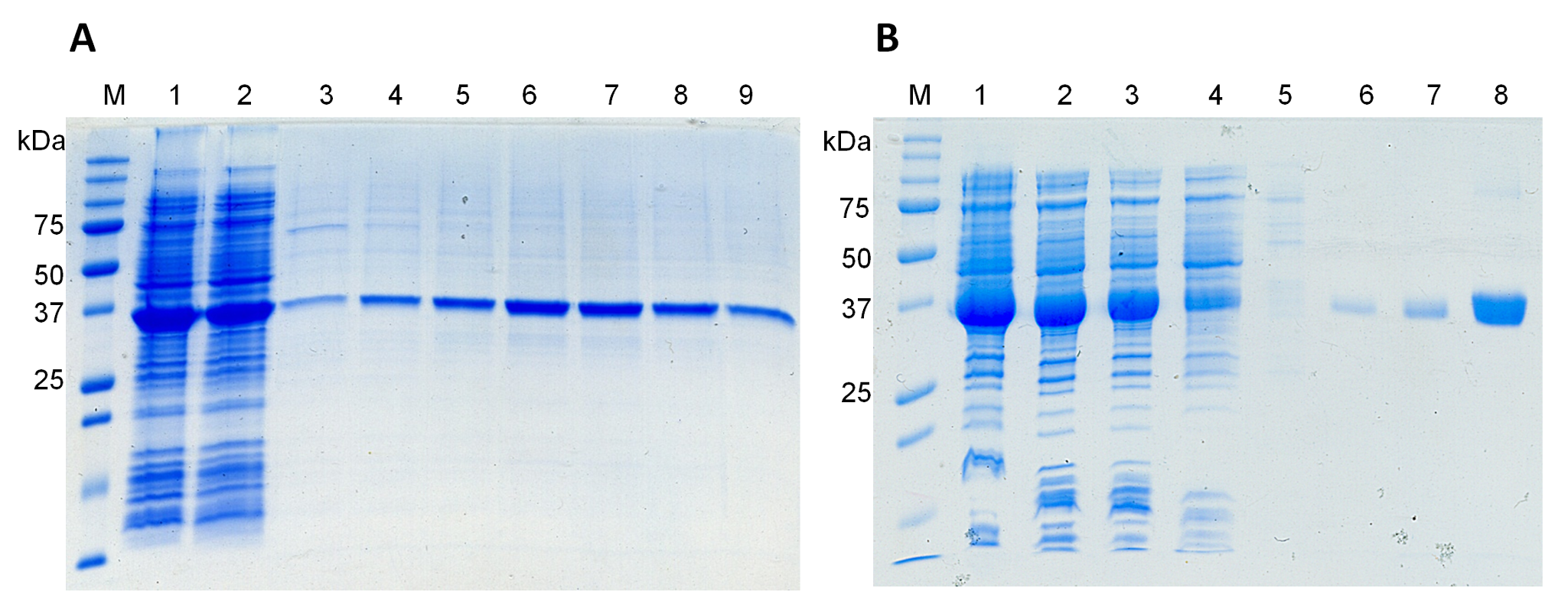

Supplement: Figure S3 — Metal-affinity purification of recombinant RS BciA and CT BciA expressed in E. coli . (A) SDS-PAGE analysis of RSBciA purification. M indicates the molecular weight marker with the corresponding weight (kDa) labeled on the left. Lane 1 shows the soluble portion of the cell lysate. Lane 2 shows contaminating proteins that did not bind to the Ni2+ affinity column, as well as excess RSBciA. Lanes 3–9 show the elution of pure ∼37 kDa RSBciA from the column in concentrations 85–220 mM imidazole. (B) SDS-PAGE analysis of CTBciA purification. M indicates the molecular weight marker with the corresponding weight (kDa) labeled on the left. Lanes 1 and 2 shows the total and soluble portion of the cell lysate. Lane 3 shows contaminating proteins that did not bind to the Ni2+ affinity column, as well as excess CTBciA. Lanes 4–7 show elution from the column with increasing concentrations of imidazole. Lane 8 shows elution of pure CTBciA in 250 mM imidazole. (TIF) [file pone.0089734.s003.tif]

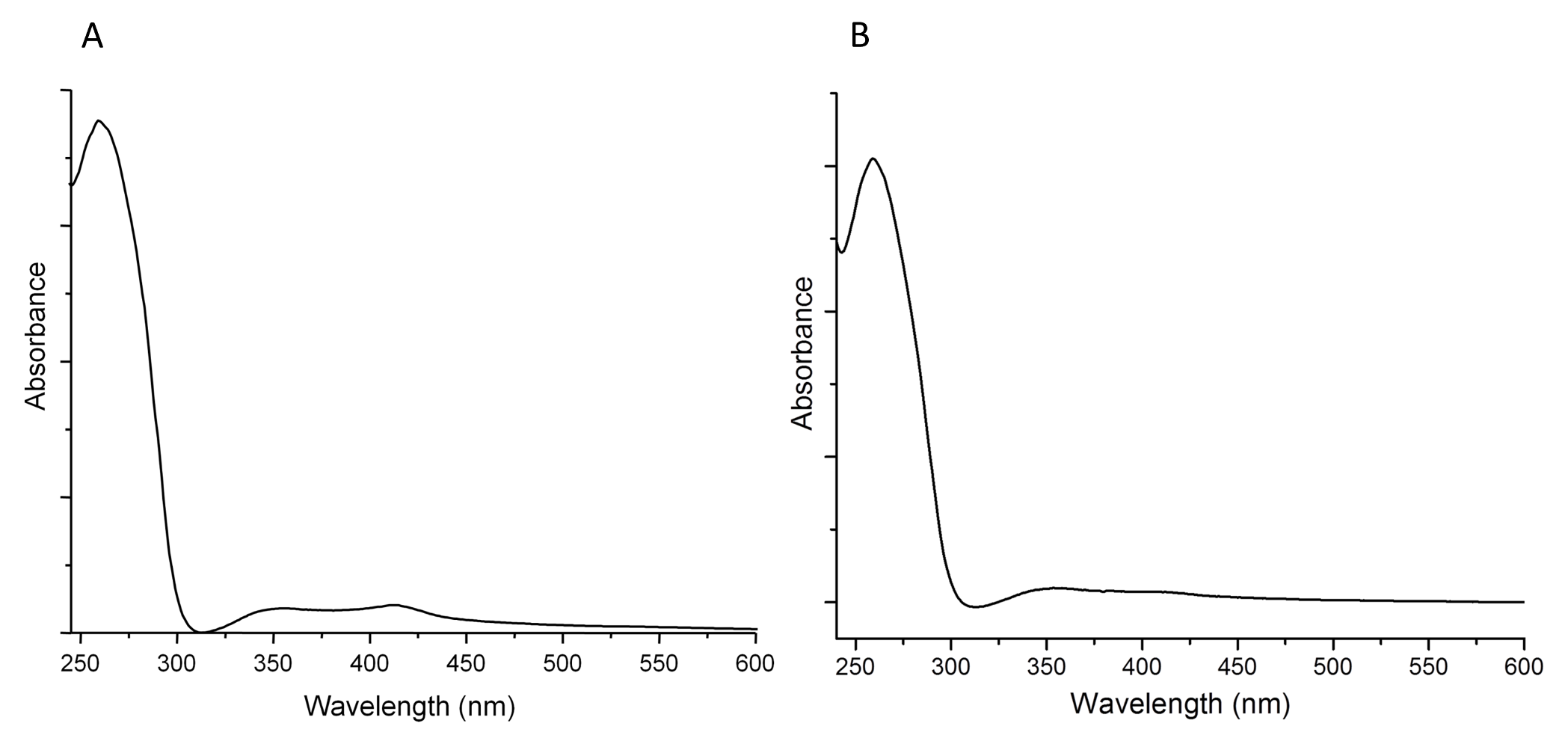

Supplement: Figure S4 — Nucleotide(s) copurify with recombinant RS BciA and CT BciA. UV/Vis wavescans of purified 8-vinyl reductases RSBciA (A) and CTBciA (B) reveal absorbance maxima at 260 nm. This suggests that an unknown nucleotide(s) co-purifies with the enzymes, likely due to the presence of the conserved and essential NADPH binding site at the N-termini of both proteins. (TIF) [file pone.0089734.s004.tif]

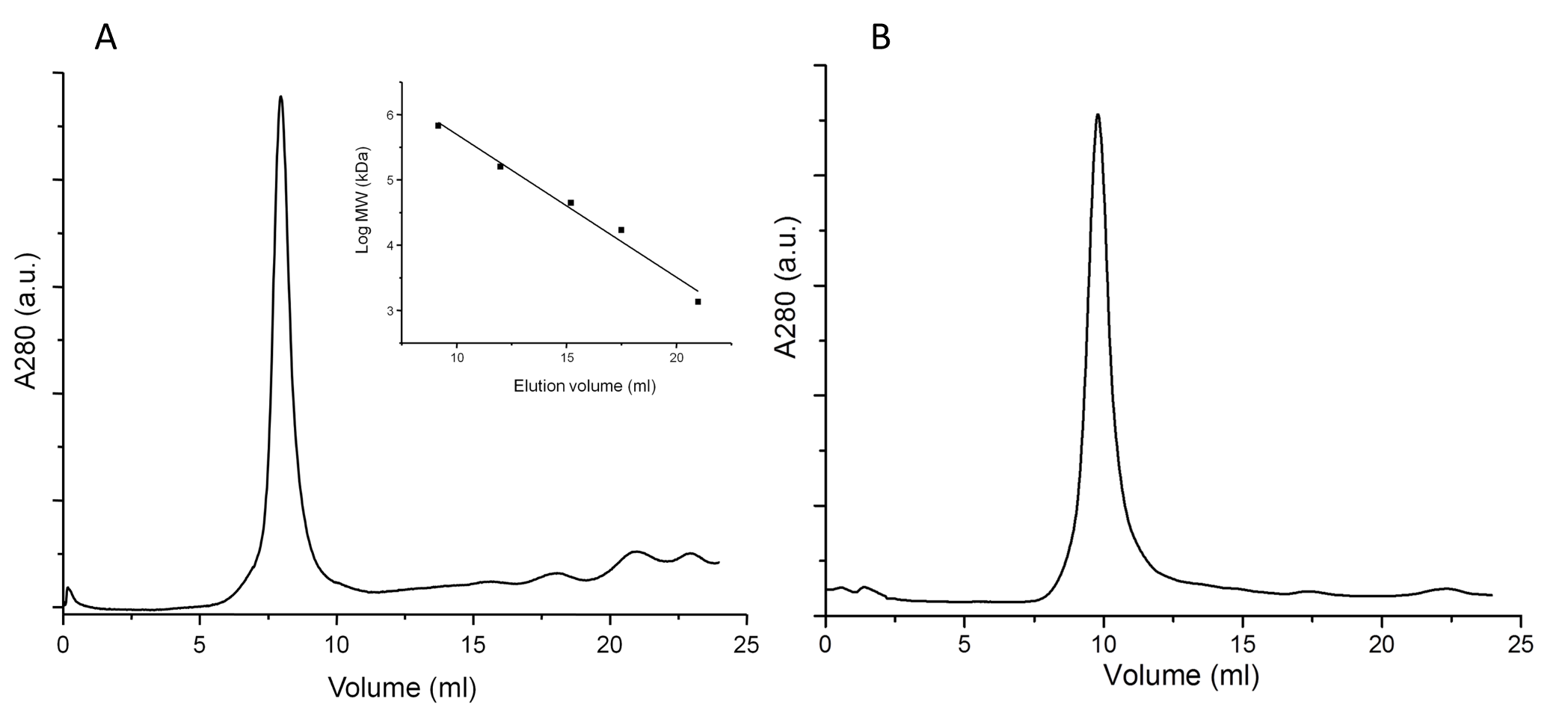

Supplement: Figure S5 — Recombinant 8-vinyl reductases behave as large complexes/aggregates in solution. (A). RSBciA elutes from a size exclusion column after 8.5 mL, close to the void volume of the column. RSBciA appears to be aggregating in solution, despite the presence of the protein stabilizing agent glycerol (10%). (B). CTBciA elutes from the same size exclusion column after 10 mL, suggesting that it is forming a large complex close to 600 kDa (as determined using protein standards of known molecular weight). (TIF) [file pone.0089734.s005.tif]

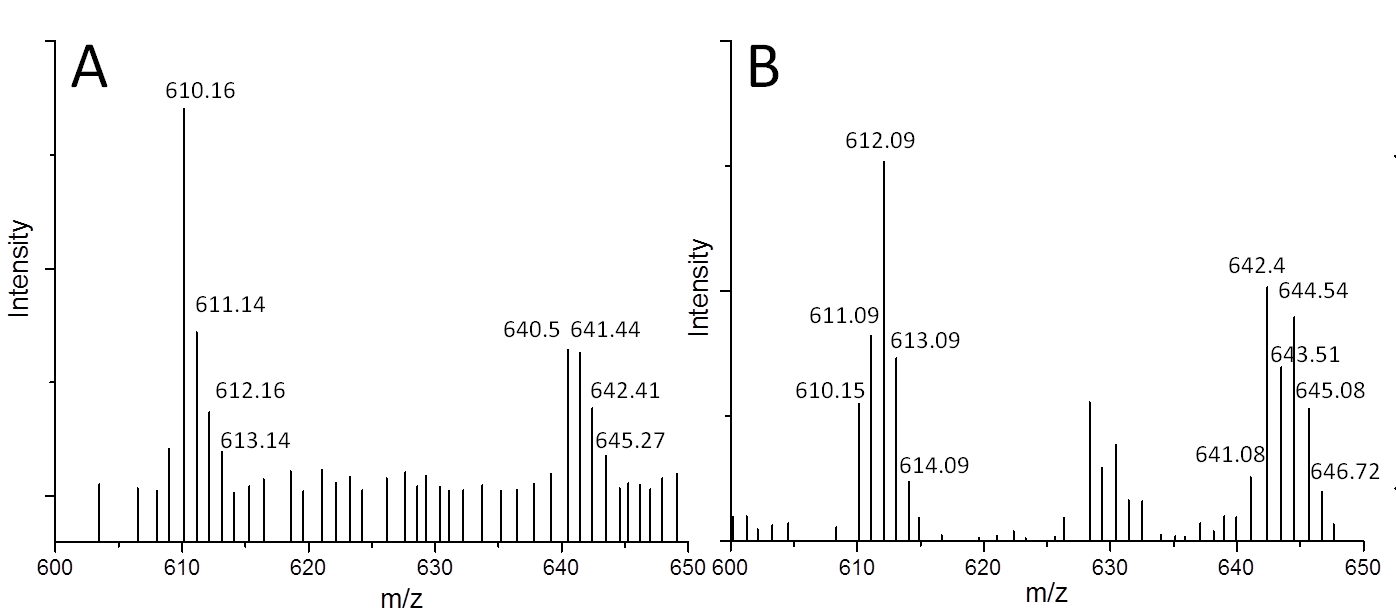

Supplement: Figure S6 — Mass spectra of divinyl-protochlorophyllide and mono-vinyl-protochlorophyllide. (A). The peak at m/z 610 is characteristic of divinyl-protochlorophyllide. (B). Upon the reduction of divinyl-protochlorophyllide by RSBciA and CTBciA to the mono-vinyl form, two protons are added and the mass shifts to m/z 612. The peaks at m/z 642 and 644, respectively, represent methanol adducts of the two compounds. (TIF) [file pone.0089734.s006.tif]
