# Supplementary material for: A Tale of Two Reductases: Extending the Bacteriochlorophyll Biosynthetic Pathway in E. coli
Source: PLoS One. 2014 Feb 21;9(2):e89734. doi: 10.1371/journal.pone.0089734 (PMC3931815; doi:10.1371/journal.pone.0089734)
Supplement: Table S1 — Primers used in this study. Restriction sites are represented in lower case. (DOCX) [file pone.0089734.s007.docx]

Table S1. Primers used in this study.

| P1 | 5’-ATAcatatgTCCGAGACCGCCCCCCTGC-3’ |
| --- | --- |
| P2 | 5’-ATgcggccgcTCAATGGTGATGGTGATGGTGACGATTTCCGGGCGATCCTTCTGC-3’ |
| P3 | 5’-ATAcatatgTCATCTTCGTCTGTACTGGCTG-3’ |
| P4 | 5’-ATgcggccgcTCAGTGGTAGTGGTAGTGGTAGAACATCGCGTGCGCGCCGAG-3’ |
| P5 | 5’-ACAGATCTAATGTCCGAGACCGCCCCCCTGC-3’ |
| P6 | 5’-ATGCGGCCGCTCAATGGTGATGGTGATGGTGACGATTTCCGGGCGATCCTTCTGC-3’ |
| P7 | 5’-CAAGATCTATGAGCAGCCCATCATTCAAC-3’ |
| P8 | 5’-ATGCGGCCGCTCAGCGGCGAGCCACG-3’ |
| P9 | 5’-atagatctATGTCATCTTCGTCTGTACTGGCTG-3’ |
| P10 | 5’-ATgcggccgcTCAGAACATCGCGTGCGCGCCGAG-3’ |
| P11 | 5’-ACagatctATGACCGCGCATGACCAGCG-3’ |
| P12 | 5’-AAgcggccgcTCAGCGCCCCTTGGGCAG-3’ |

Restriction sites are represented in lower case.
