# Supplementary material for: A Tale of Two Reductases: Extending the Bacteriochlorophyll Biosynthetic Pathway in E. coli
Source: PLoS One. 2014 Feb 21;9(2):e89734. doi: 10.1371/journal.pone.0089734 (PMC3931815; doi:10.1371/journal.pone.0089734)
Supplement: Table S2 — Plasmids used in this study. (DOCX) [file pone.0089734.s008.docx]

**Table S2. Plasmids used in this study.**

| **Plasmid** | **Relevant properties** | **Source** |
| --- | --- | --- |
| pACmod-*hemABCD* | Constitutive expression of *R. capsulatus hemA* and *E. coli hemB, hemC,* and *hemD* | [[1](#_ENREF_1)] |
| pBBR-*hemEF* | Constitutive expression of *Synechocystis hemE* and *E. coli hemF* | [[1](#_ENREF_1)] |
| pUCmod-*bchDIS* | Constitutively expression of *C. tepidum* *bchD*, *bchI* and *bchS* | [[2](#_ENREF_2)] |
| pCDFBB | Empty pCDFBB (BioBrick^Tm^) vector | Choudhary, S. and Schmidt-Dannert, C., unpublished |
| pCDFBB-*bchM* | Constitutive expression of *C. tepidum* *bchM* | This paper |
| pCDFBB-*rsbciA* | Constitutive expression of *R. sphaeroides* *bciA* | This paper |
| pCDFBB-*ctbciA* | Constitutive expression of *C. tepidum* *bciA* | This paper |
| pCDFBB-*bchM-rsbciA* | Constitutive expression of *C. tepidum* *bchM* and *R. sphaeroides* *bciA* | This paper |
| pCDFBB-*bchM-ctbciA* | Constitutive expression of *C. tepidum* *bchM* and *bciA* | This paper |
| pCDFBB-*bchJ* | Constitutive expression of *R. sphaeroides* *bchJ* | This paper |
| pCDFBB-*bchM-bchJ* | Constitutive expression of *C. tepidum* *bchM* and *R. sphaeroides* *bchJ* | This paper |
| pCDFBB-*bchM-rsbciA-bchJ* | Constitutive expression of *C. tepidum* *bchM* and *R. sphaeroides* *bciA and bchJ* | This paper |

**SUPPORTING REFERRENCES**

1. Kwon SJ, de Boer AL, Petri R, Schmidt-Dannert C (2003) High-level production of porphyrins in metabolically engineered *Escherichia coli*: systematic extension of a pathway assembled from overexpressed genes involved in heme biosynthesis. Appl Environ Microbiol 69: 4875-4883.

2. Johnson ET, Schmidt-Dannert C (2008) Characterization of three homologs of the large subunit of the magnesium chelatase from *Chlorobaculum tepidum* and interaction with the magnesium protoporphyrin IX methyltransferase. J Biol Chem 283: 27776-27784.
